# A genetically defined tecto-thalamic pathway drives a system of superior-colliculus-dependent visual cortices

**DOI:** 10.1016/j.neuron.2023.04.022

**Published:** 2023-05-11

**Authors:** Joshua M. Brenner, Riccardo Beltramo, Charles R. Gerfen, Sarah Ruediger, Massimo Scanziani

**Affiliations:** 1 Department of Physiology, University of California, San Francisco, San Francisco, CA, USA; 2 Howard Hughes Medical Institute, University of California, San Francisco, San Francisco, CA, USA; 3 University of Cambridge, Cambridge, UK; 4 National Institute of Mental Health, Bethesda, MD, USA

## Abstract

Cortical responses to visual stimuli are believed to rely on the geniculo-striate pathway. However, recent work has challenged this notion by showing that responses in the postrhinal cortex (POR), a visual cortical area, instead depend on the tecto-thalamic pathway, which conveys visual information to the cortex via the superior colliculus (SC). Does POR’s SC-dependence point to a wider system of tecto-thalamic cortical visual areas? What information might this system extract from the visual world? We discovered multiple mouse cortical areas whose visual responses rely on SC, with the most lateral showing the strongest SC-dependence. This system is driven by a genetically defined cell type that connects the SC to the pulvinar thalamic nucleus. Finally, we show that SC-dependent cortices distinguish self-generated from externally generated visual motion. Hence, lateral visual areas comprise a system that relies on the tecto-thalamic pathway and contributes to processing visual motion as animals move through the environment.

## Introduction

The mammalian visual cortex receives visual signals from at least two separate pathways. One, the geniculo-striate pathway, links the retina to the dorsolateral geniculate nucleus (dLGN) of the thalamus, which projects to the visual cortex. The other, the tecto-thalamo-cortical pathway, links the retina to the visual cortex via the superior colliculus (SC)^1,2^ and is highly conserved across species. This tecto-thalamo-cortical pathway consists of two branches, one through the pulvinar nucleus of the thalamus and the other one through the dLGN. The tecto-pulvino-cortical branch was first postulated by Diamond and Hall^3^ to be the ancestral route of visual information to the cortex and was recently demonstrated anatomically and physiologically. Specifically, studies in primates^4–6^ have indicated that pulvinar neurons receiving input from the superficial (visual) layers of the SC project to a visual cortical area referred to as middle temporal (MT), or to areas surrounding MT.^7^ Furthermore, in rodents, a disynaptic pathway between the SC and visual cortex via the pulvinar (also referred to as the lateral posterior nucleus; LP^8^) has been recently confirmed.^9,10^ In contrast to the geniculo-striate pathway, considered the main conduit of visual information to the visual cortex, however, SC lesions and silencing experiments suggest a minor and feature-selective contribution of the tecto-thalamo-cortical pathway to cortical visual responses in primates,^11–13^ cats,^14^ and rodents.^15,16^ For example, lesions of the SC in primates have little effect on the response of area MT to visual stimuli^11,12^ unless the primary visual cortex (V1) had been previously ablated.^11,17^ In contrast to these observations, recent work in the mouse visual system has revealed that visual responses in a visual cortical area called the postrhinal cortex (POR) rely almost entirely on the tecto-thalamo-cortical pathway^9^ and not on the geniculo-striate pathway. Is POR an idiosyncrasy within the visual cortex of the mouse in its dependence on SC, or does it point to a larger system of cortical areas dedicated to processing visual information from the SC? Furthermore, is the tecto-pulvinar pathway the main conduit of visual information from the SC to the cortex? Finally, POR is particularly responsive to moving visual stimuli, a property that it inherits from the SC.^9,18^ However, such visual motion is fundamentally ambiguous as it can be generated either by movement of external objects or else self-generated as the animal traverses its environment. Do SC-dependent visual cortical areas and the SC itself disambiguate between these two types of motion?

Here, we combined transsynaptic anterograde viral tracing with widefield calcium imaging to identify a lateromedial gradient of visual cortical areas subordinate to the SC. The most lateral of these cortices rely almost entirely on the SC for their visually evoked activity, a bias that diminishes moving medially across the cortex. By perturbing a genetically defined population of SC neurons that project to the pulvinar nucleus of the thalamus, we identify the main conduit of visual information to these SC-dependent cortices. Finally, we trained head-fixed mice to drive the movement of a visual stimulus by running on a treadmill and discovered that SC-dependent cortices respond preferentially to externally generated motion. This study thus reveals a tecto-thalamic pathway through which the SC drives visual responses along a lateromedial gradient across the mouse visual cortex and identifies a potential role of this pathway in distinguishing self from externally generated motion. Together, these lateral cortices constitute a functionally distinct tecto-thalamic visual cortical system operating in parallel with the more medial canonical geniculo-striate system.

## Results

### A lateromedial gradient of SC-driven activity in the visual cortex

**Figure 1:**
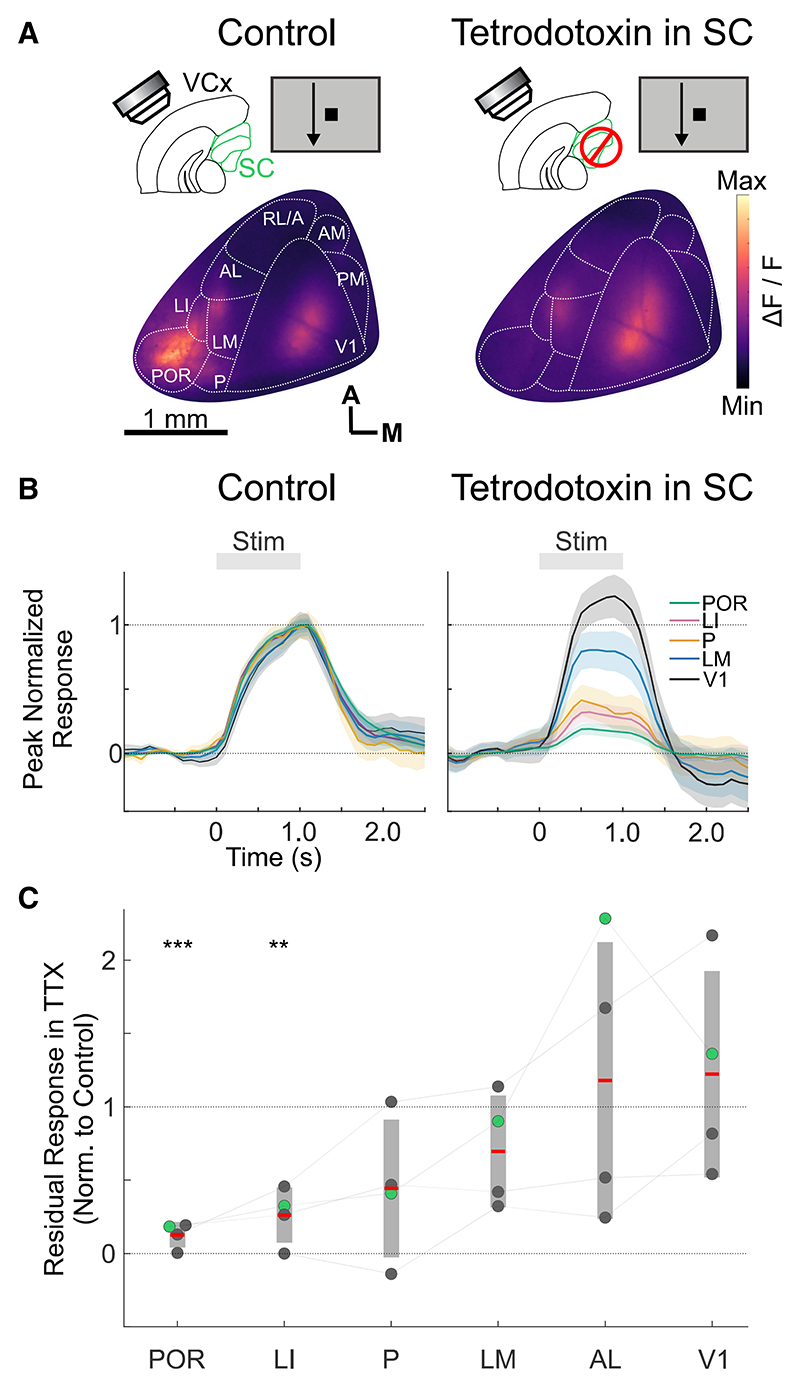
A lateromedial gradient of SC-dependent HVAs (A) Top: schematic of experimental configuration for widefield calcium imaging of visual cortex (VCx) in response to stimuli consisting of moving dots under control condition (left) and tetrodotoxin (TTX) silencing of superior colliculus (SC; right). Bottom: example ΔF/F (120 trial average) across visual areas before (left) and after (right) TTX injection in SC. Cortical visual areas delineated as shown in Figure S1. POR, postrhinal cortex; LI, laterointermediate area; P, posterior area; LM, lateromedial area; AL, anterolateral area; AM, anteromedial area; PM, posteromedial area; RL/A, rostrolateral/anterior areas; V1, primary visual cortex. Orientation: A, anterior; M, medial. (B) Time courses of calcium response to visual stimulus from the example experiment above. Traces are normalized to peak response in each area under control condition. Shading: SEM. (C) Summary data for 4 mice. y axis plots remaining visual response after TTX injection in SC, normalized by the control response for each area. Gray bar shows 95% confidence interval above and below mean (red line). Gray lines between dots link areas from the same animal. p values are calculated with paired t test. Green data are from the example mouse shown in (A) and (B).

To identify visual areas whose response to visual stimuli depends on the SC, we surveyed the occipital cortex of head-fixed mice expressing GCaMP6s in pyramidal neurons^19^ under the lens of a widefield microscope. After generating retinotopic maps to delineate V1 and higher visual areas (HVAs; Figures S1 and S2; see STAR Methods), we presented a visual stimulus consisting of a 2° dark square dot moving at 30°/s on a computer monitor placed on the contralateral side to the imaged hemisphere (Figures 1A and 1B). This stimulus was chosen because of the robust responses it elicits in the SC.^18^ Although several cortical visual areas responded to the moving dot, including V1 and HVAs lateral to V1, silencing the ipsilateral SC with tetrodotoxin (TTX) selectively impaired responses in three lateral HVAs (Figure 1C). These areas included POR (87.2% reduction; N = 4 mice; p < 10^−3^), consistent with previous results, as well as the laterointermediate area (LI) (73.8% reduction; N = 4 mice; p < 0.01) and posterior area (P) (55.7% reduction; N = 4 mice; p < 0.1). Responses in the lateromedial area (LM) also trended toward a reduction, with a smaller effect size (30.4% reduction; N = 4 mice; p = 0.22). In contrast, visual responses in V1 and anterolateral area (AL) remained, on average, nearly unchanged (V1: 22% increase; N = 4 mice; Not signficant [NS], AL: 18% increase; N = 4 mice; NS), indicating that the effect of SC silencing on lateral HVAs was not simply due to a general reduction in visually evoked cortical activity.

These results show that while three lateral HVAs mostly depend on SC for their visual response to moving dots, other areas, including V1, are largely independent of this midbrain structure. The impact of SC silencing was not stimulus specific. In fact, responses to patches of drifting gratings, i.e., the visual stimuli used to delineate HVAs (see STAR Methods), were impaired by SC silencing in those lateral HVAs whose responses to moving dots were also reduced (Figure S3; POR: 74.2% reduction; N = 4 mice; p < 10^−3^. LI: 66.0% reduction; N = 4 mice; p < 10^−3^. P: 60.5% reduction; N = 4 mice; p < 0.01. LM: 38.7% reduction; N = 4 mice; p < 0.05. Posteromedial area [PM]: 36.2% reduction; N = 4 mice; p < 0.05. AL: 28.2% reduction; N = 4 mice; NS. V1: 25.7% reduction; N = 4 mice; NS. Anteromedial area [AM]: 26.1% reduction; N = 4 mice; NS). These data reveal the presence of a lateromedial gradient across cortical visual areas relative to the reliance on the SC as a source of visual input, with the lateral areas being most affected by SC silencing.

**Figure 2:**
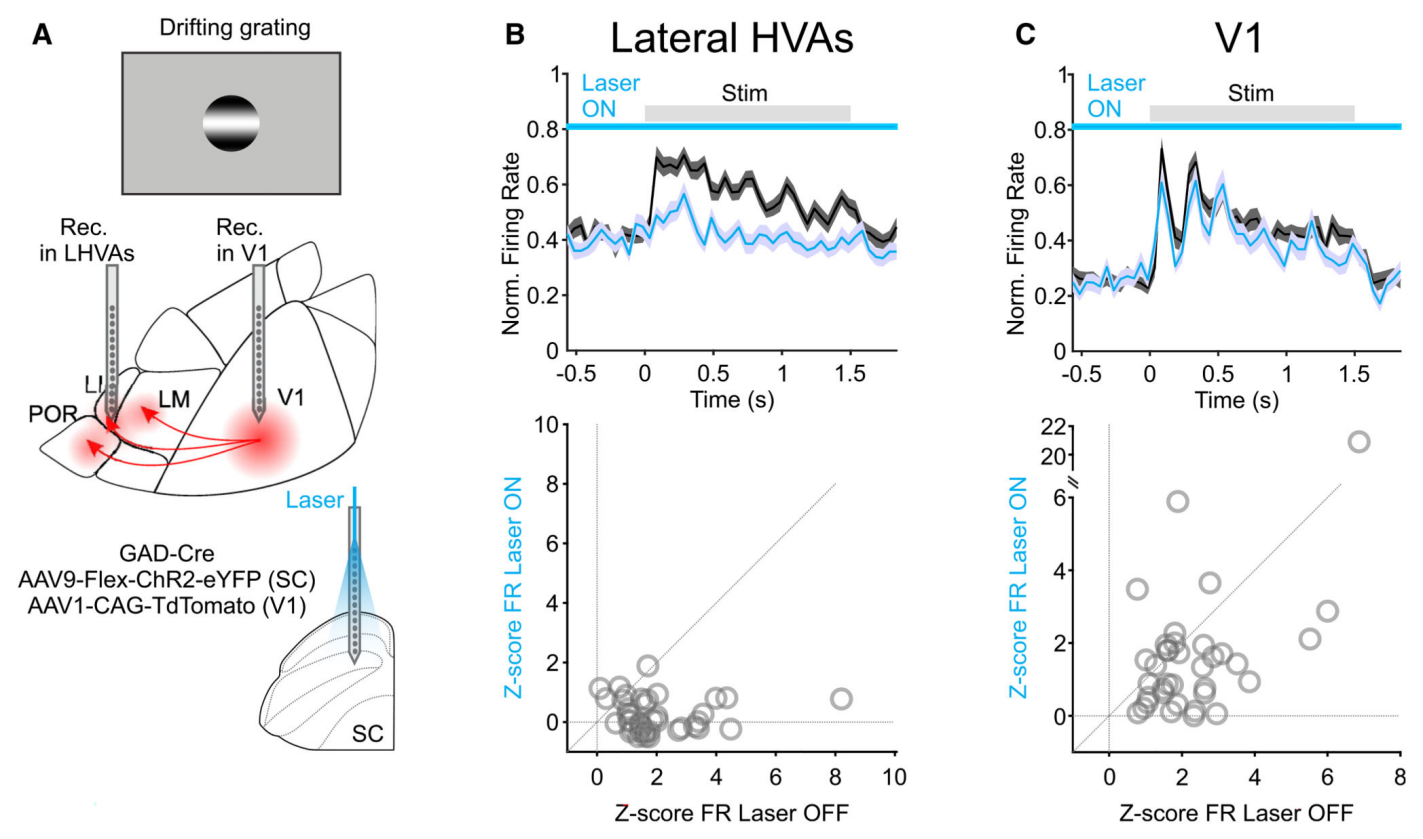
Visual responses of isolated units in lateral HVAs depend on SC (A) Schematics of experimental configuration. An optrode is used to silence neuronal activity in SC by optogenetically activating GABAergic neurons conditionally expressing ChR2 in a GAD-Cre mouse. Electrophysiological recordings are performed simultaneously in visuotopically matching regions of V1 and lateral HVAs. The visual stimulus consists of a drifting grating (diameter: 45°). The recordings in the lateral HVAs were centered around LI near its borders with POR and LM. The stereotactic injection of the anterograde tracer AAV1.CAG.tdTomato (red) in V1 enabled the identification of the areas LI, POR and LM. (B) Summary Peri-stimulus time histogram (PSTH) for 42 visually responsive isolated regular spiking (RS) units recorded in lateral HVAs centered around LI of awake head-fixed mice in response to visual stimulation under control conditions (black) and during SC optogenetic silencing (blue). The gray horizontal bar illustrates the duration of stimulus presentation and the blue horizontal bar indicates the period of SC silencing. Bottom: scatterplot for the isolated units (firing rate [FR] average *Z* scores ± SEM: laser OFF: 1.98 ± 0.22; laser ON: 0.21 ± 0.09; p < 10^−7^, n = 42 RS units, N = 4 mice, Wilcoxon signed-rank test). Data plotted as mean ± SEM (shaded area). (C) Top: as in (B), but for 36 visually responsive isolated RS units recorded in the V1 simultaneously with the recordings in lateral HVAs shown in (B). Bottom: scatterplot for the isolated units (FR average *Z* scores ± SEM: laser OFF: 2.29 ± 0.23; laser ON: 1.93 ± 0.58 ; p = 0.005, n = 36 RS units, N = 3 mice, Wilcoxon signed-rank test).Data plotted as mean ± SEM (shaded area).

This gradient of SC-dependency was confirmed electrophysiologically by recording from V1 and lateral HVAs while optogenetically silencing the SC. The recordings in lateral HVAs were centered around LI near its borders with POR and LM, and visuotopically matched with an SC recording site. The electrode inserted in the SC was coupled to an optic fiber for optogenetic silencing and simultaneous monitoring of SC activity (see STAR Methods; Beltramo and Scanziani^9^). In three out of four mice, we simultaneously recorded from lateral HVAs and visuotopically matched regions in V1. We silenced the SC on inter-leaved trials by activating glutamic acid decarboxylase (GAD)-Cre neurons conditionally expressing Channelrhodopsin2 (ChR2), and quantified the effect of SC silencing on lateral HVAs and V1 visual responses. SC silencing reduced responses to patches of drifting gratings in lateral HVAs significantly more than in V1 (p < 10^−3^), thus consistent with our widefield imaging data (Figure 2; lateral HVAs: 77.7% mean reduction, p < 10^−7^, n= 42 regular spiking units, N = 4 mice; V1: 28.7% mean reduction, p < 0.05, n = 36 regular spiking units, N = 3 mice) and with the presence of a lateromedial gradient of SC-dependency across the visual cortex.

### A genetically defined tecto-pulvinar pathway underlies SC-driven activity in visual cortex

**Figure 3:**
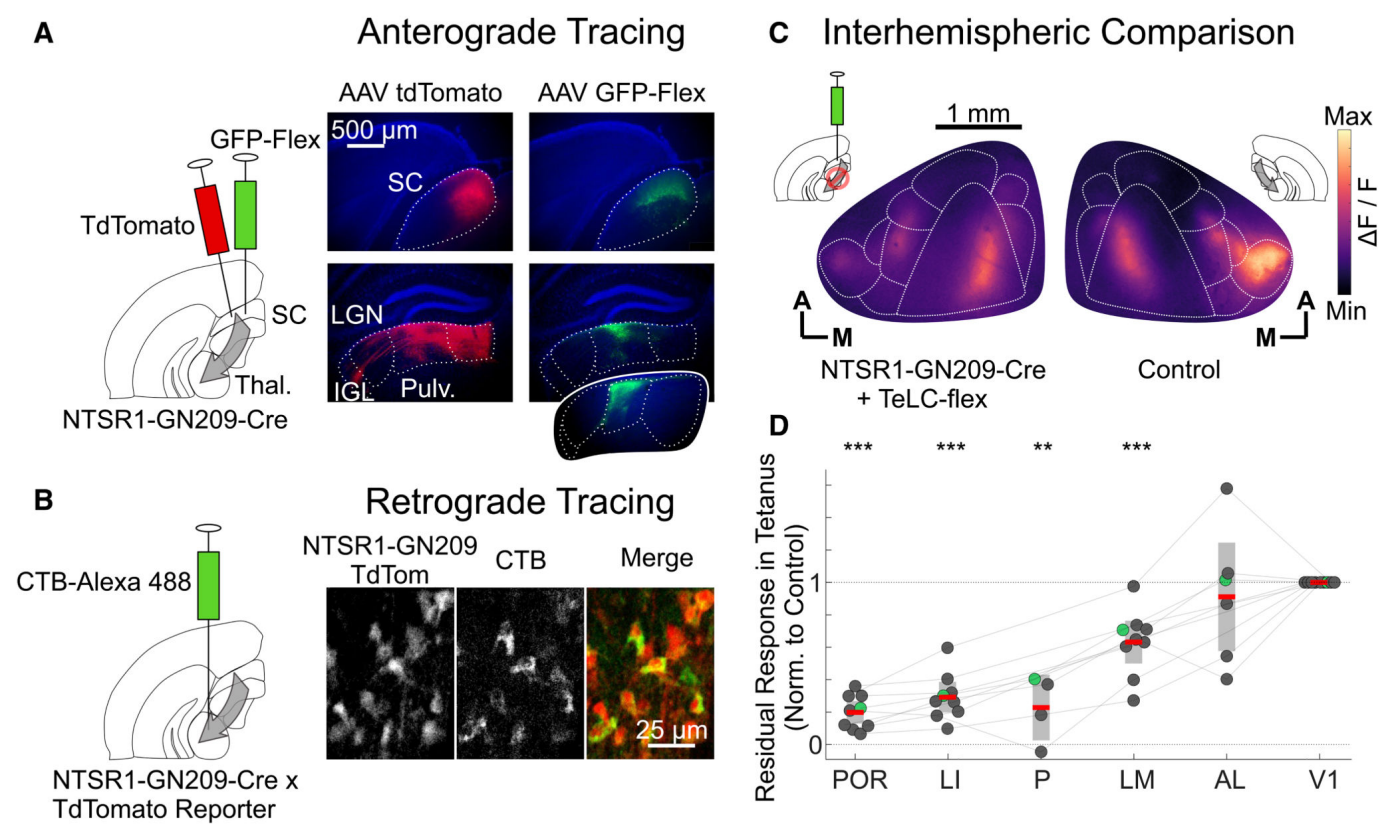
A genetically defined cell type in SC mediates visual responses in lateral visual areas (A) Left: schematic of anterograde tracing with injection of AAV-tdTomato and AAV-GFP-flex in SC of NTSR1-GN209-Cre mouse to label projections from SC to thalamus. Right: photomicrographs of the same two coronal sections (bregma: –2.1 mm) showing tdTomato (left) and GFP (right) expression at injection site (top) and along thalamic projections (bottom). Note the restricted projection pattern of the NTSR1-GN209-Cre expressing SC neurons to pulvinar (GFP) as compared with the broader tecto-thalamic projection pattern captured by the nonspecific expression of tdTomato in SC. Inset: photomicrograph from a more caudal section (bregma −2.5 mm) illustrating the stronger tecto-pulvinar projection in more caudal regions of the pulvinar. Dotted lines delineate various brain structures according to the Allen Brain Atlas. LGN, lateral geniculate nucleus; Pulv, pulvinar nucleus; IGL, intergeniculate leaflet. (B) Left: schematic of retrograde labeling of pulvinar-projecting SC neurons with CTB-Alexa-488 in NTSR1-GN209-Cre mouse crossed with tdTomato reporter. Actual injection site is 1.25 mm anterior to the plane depicted in the schematic. Right: confocal images showing expression of tdTomato reporter in SC (left panel), retrograde uptake of CTB conjugate (middle panel), and composite of the two (right panel). (C) Top: schematics of experimental configuration for widefield calcium imaging of visual cortex from the hemisphere in which ipsilateral SC conditionally expresses TeLC in NTSR1-GN209-Cre-tagged neurons (left) and from the control hemisphere (right). Bottom: example ΔF/F (240 trial average) across visual areas in TeLC (left) and control (right) hemispheres. Cortical visual areas delineated as shown in Figure S1. Orientation: A, anterior; M, medial. (D) Summary data: response magnitude for each area in TeLC hemisphere relative to the response in the contralateral hemisphere. Within each hemisphere, responses were normalized by the response in their respective V1 (see STAR Methods). Gray bar shows 95% confidence interval above and below mean (red line). Gray lines between dots link areas from the same animal. p values are calculated with a t test. Green dots represent data from the example mouse shown in (C). Within each mouse, data on the impact of TeLC on a given area was only included if that area responded to the visual stimulus in the control hemisphere (see STAR Methods).

The tecto-pulvinar pathway is a tecto-thalamic route that disynaptically links the SC to HVAs via the pulvinar.^9,10^ What is the contribution of this pathway to visual responses in SC-dependent HVAs? To address this question, we first needed to identify a mouse line that would allow us to selectively and completely access the tecto-pulvinar pathway. The NTSR1-GN209-Cre mouse line is a candidate because it tags those SC neurons projecting to the pulvinar and not SC neurons projecting to other thalamic nuclei; for example, the dLGN^18,20,21^ (Figure 3A). What fraction of the pathway linking the SC to the pulvinar is composed of axons originating from NTSR1-GN209 neurons? We crossed the NTSR1-GN209-Cre line with the Ai14 tdTomato reporter and injected the retrograde tracer Cholera Toxin B (CTB) in the pulvinar. The large majority of neurons projecting from SC to the pulvinar, i.e. CTB-labeled neurons, expressed TdTomato (mean ± standard error of the mean [SEM]: 85.20 ± 1.529% co-labeled; N = 3 mice) (Figure 3B), indicating that the NTSR1-GN209 line is suitable for a selective and almost complete perturbation of the tecto-pulvinar pathway. We determined the contribution of the tecto-pulvinar pathway to visual response in HVAs by blocking synaptic transmission in NTSR1-GN209-Cre-tagged neurons through the conditional expression of tetanus light chain (TeLC^22^). We restricted the injection of TeLC-encoding adeno associated virus (AAV) to the left SC (Figure S4; viral spread, mean ± SEM: medio-lateral: 1.285 ± 0.056 mm; spatial coverage: 78.519 ± 1.958%; anterior-posterior: 1.320 ± 0.108 mm; spatial coverage: 80.589 ± 6.619%, N = 6 mice) and compared visual responses between the left and right cortical hemispheres at least 2 weeks after the injection. The extent of the viral injection was confirmed histologically (see STAR Methods; Figure S4). To compare activity in HVAs between hemispheres, their visual responses were normalized to the average V1 response measured within the same hemisphere. Selective expression of TeLC in the tecto-pulvinar pathway nearly abolished responses to moving dots in POR, P, and LI, and significantly reduced responses in LM (Figures 3C and 3D; POR: mean reduction 80.14%; N = 9 mice; p < 10^−6^. LI: mean reduction: 70.78%; N = 9 mice; p < 10^−4^. P: mean reduction = 77.19%; N = 4 mice; p < 0.01. LM: mean reduction: 36.82%; N = 9 mice; p < 10^−4^). In contrast, responses in the more medial area AL were, on average, spared (AL: mean reduction: 8.87%; N = 6 mice; NS). The responses to grating stimuli used to delineate HVAs, however, were less affected than those to moving dots, suggesting some stimulus specificity inthe projection of NTSR1-GN209-Cre-tagged neurons to the thalamus (Figure S5). These results thus demonstrate that responses to moving dots are conveyed to HVAs via the NTSR1-GN209-Cre-tagged neurons.

The significant yet incomplete reduction of visual responses in LM by TeLC expression in the SC (as compared with the POR, LI, and P; see above) suggests that visual responses in LM, in addition to being mediated by the tecto-pulvinar pathway, may also be inherited from the geniculo-striate pathway. That is, LM may represent a visual area at the intersection of the two visual pathways. If so, silencing V1 should also result in only a partial reduction in visual responses in LM. To test the extent of LM’s dependence on V1, we optogenetically silenced V1^23^ (see STAR Methods) while recording extracellular activity in LM using a silicon probe. To maximize the efficiency of the photoinhibition (see STAR Methods), we ensured a precise alignment between the optogenetically silenced area in V1 and the retinotopically matched area in LM where the visual responses were recorded (Figure S6). Abolishing visual responses in V1 (see STAR Methods), significantly but incompletely reduced visual responses in LM (LM: reduction = 44.89%; n = 24 regular spiking cells, N = 4 mice; p < 0.001). Thus, LM is a visual area that inherits visual responses from both the tecto-pulvinar and the geniculo-striate pathways. Taken together, these experiments delineate a lateromedial gradient of tecto-pulvinar dependence of visual responses across the mouse visual cortex.

### A lateromedial gradient of tecto-pulvinar versus V1 afferent input

**Figure 4:**
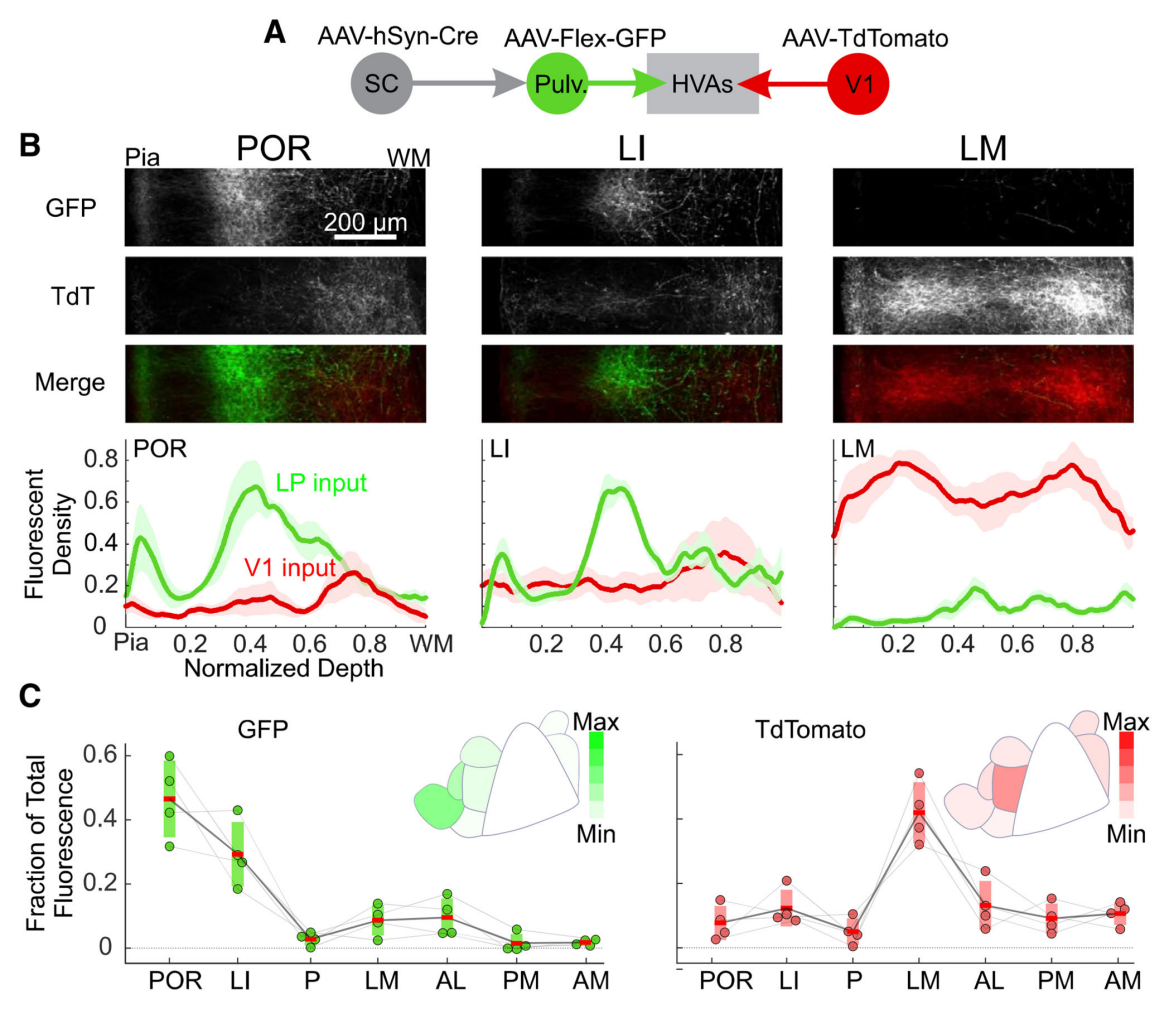
A lateromedial gradient of tecto-pulvinar versus V1 afferent input (A) Schematic of experimental configuration. V1 projections to HVAs are labeled with tdTomato, while SC projections to HVAs via pulvinar are labeled with GFP using transsynaptic anterograde tracing. (B) Top: confocal fluorescent micrograph of coronal sections of three example HVAs (POR, LI and LM) illustrating tecto-recipient pulvinar afferents (GFP; top row), V1 afferents (TdTomato; middle row), and the two merged in a composite image (bottom row). Pial surface (Pia) and white matter (WM) are to the left and right of the micrographs, respectively. Bottom: summary data. Average normalized depth profiles of fluorescent density across all mice (N = 4 mice). Each fluorophore is normalized to the maximum density across all areas of a given mouse. Data plotted as mean ± SEM (shaded area). (C) Fraction of total GFP (left) and tdTomato (right) fluorescence measured with each area. Bars show 95% confidence intervals above and below mean (black line). Gray lines between dots link areas from the same animal. Insets present anatomical “heat-maps” with average fraction of total fluorescence in each area.

Although all delineated HVAs receive direct input from V1,^24^ the above results show that their reliance on SC varies dramatically depending on their lateromedial position. We may thus expect a lateromedial gradient in the ratio between afferents of tecto-pulvinar origin and afferents originating from V1. To compare the magnitude of the two afferent inputs within each HVA, we combined two distinct anterograde labeling techniques, one to label V1 afferents and the other to specifically label pulvinar afferents belonging to the tecto-pulvinar pathway. V1 neurons were labeled by injecting AAV encoding tdTomato into V1. We selectively labeled pulvinar neurons receiving SC input with GFP, using an anterograde transsynaptic intersectional viral approach^9,25^ (Figure 4A). The collected brain tissue sections were imaged using scanning confocal microscopy, and the relative intensity of fluorophores was quantified in each visual area to determine the fraction of tecto-pulvinar and V1 input (Figures 4B–4D). The lateral areas POR and LI received the largest fraction of tecto-pulvinar input (mean fraction ± SEM: POR: tecto-pulvinar: 36.71 ± 1.18%; V1: 14.36 ± 3.45%. LI: tecto-pulvinar: 25.53 ± 6.78%; V1: 19.23 ± 7.10%; N = 4 mice) followed by LM and AL (LM: tecto-pulvinar: 7.69 ± 2.34%. AL: tecto-pulvinar: 8.84 ± 3.91%; N = 4 mice). LM received the largest fraction of V1 input followed by AL (mean fraction ± SEM: LM: V1: 64.30 ± 5.60%. AL: V1: 12.58 ± .34%; N = 4 mice). Medial areas AM and PM were dominated by V1 afferents and received little to no input from pulvinar (mean fraction ± SEM: PM:tecto-pulvinar: 1.71 ± 1.72%; V1: 14.23 ± 5.05%. AM: tecto-pulvinar:1.65 ± 0.63%; V1: 11.95 ± 0.0308; N = 4 mice). Area P received a relatively small fraction of inputs from both the tecto-pulvinar and V1 pathways (mean fraction ± SEM: P: tecto-pulvinar: 2.23% ± 0.86%;V1: 4.96 ± 1.73%; N = 4 mice). Thus, the relative magnitude of tecto-pulvinar and V1 input to HVAs follows a lateromedial gradient consistent with the functional dependence of these areas on the SC.

### SC and SC-dependent HVAs distinguish self from externally generated motion in the visual scene

**Figure 5:**
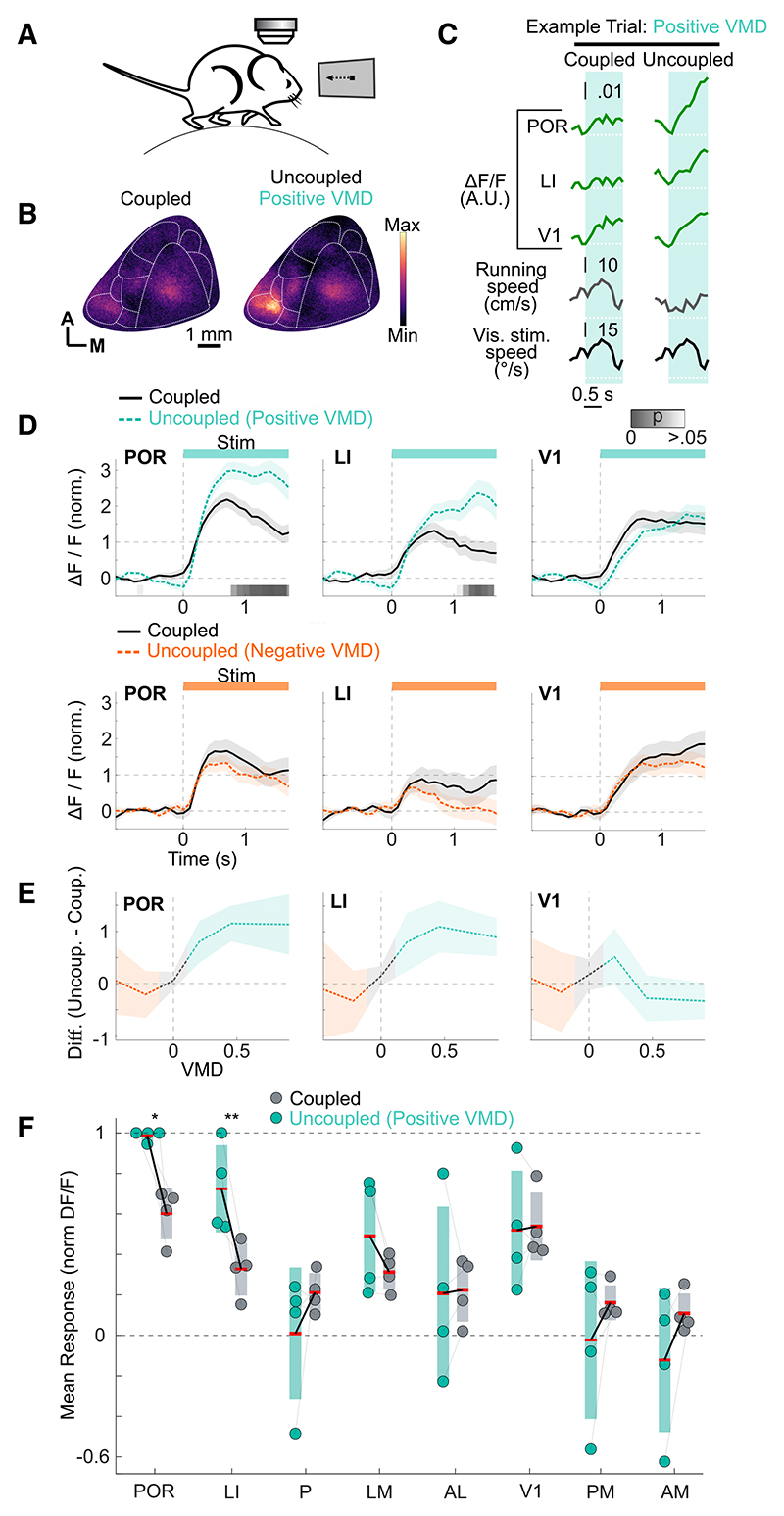
SC-dependent HVAs distinguish self from externally generated motion of visual stimuli (A) Schematic of experimental configuration for widefield calcium imaging of visual cortex in a mouse running on a treadmill. The animal is presented with a dot which progresses along a naso-temporal trajectory either at a speed commensurate to the running speed of the mouse (coupled), or, on alternate trials, with a dot that replays the previous coupled stimulus irrespective of the running speed of the mouse (uncoupled). (B) Example ΔF/F (averaged over 37 trials) across visual areas in alternated coupled (left) and uncoupled (right) presentations of visual stimulus presentations with positive visuo-motor divergence (VMD). The visual stimulus moved at the same speed in both conditions. Note the increased response of lateral visual areas in the uncoupled trials with positive visuo-motor divergence. Cortical visual areas delineated as shown in Figure S1. Orientation: A, anterior; M, medial. (C) Example trial illustrating the calcium response (ΔF/F), running speed and the moving dot speed for a pair of coupled and uncoupled stimulus presentations from a trial with positive visuo-motor divergence. The horizontal dotted lines indicate baseline for ΔF/F and 0 for running and stimulus speed. The positive visuo-motor divergence elicits a larger calcium response compared with an identical yet coupled visual stimulus in SC-dependent visual areas but not in V1. A.U.: Arbitrary units. (D) Time courses of ΔF/F for three example areas (POR, LI, and V1) averaged across all mice (N = 4 mice) for coupled (black) and uncoupled trials with positive (top row; aqua) or negative (bottom row; orange) visuo-motor divergence. Note the increased response of POR and LI in the uncoupled trials with positive visuo-motor divergence, but not in uncoupled trials with negative visuo-motor divergence. Also, note similar responses in V1 for coupled and uncoupled trials across both conditions. Responses are normalized to the average response in V1 across all coupled trials. p values are calculated with Benjamini-Hochberg method to control false-discovery rate from multiple comparisons. Data plotted as mean ± SEM (shaded area). (E) Average difference in ΔF/F between uncoupled and coupled. Vertical dotted line indicates no visuo-motor divergence, whereas values to the right and the left indicate positive (aqua) and negative (orange) visuo-motor divergence trials, respectively. (F) Summary data: ΔF/F in each area across all animals for coupled (gray) and uncoupled trials with positive visuo-motor divergence (aqua). Responses are normalized by maximal response across all areas in a given mouse. Bars show a 95% confidence interval above and below the mean (red line). Gray lines between dots link responses from the same animal between conditions. p values are calculated from a paired t test.

Is this system of SC-dependent cortical visual areas tuned to specific features of the visual world? Both the SC and the SC-dependent cortex show robust responses to the movement of objects through the visual scene.^9,18^ However, such motion is fundamentally ambiguous. In fact, the displacement of an object across the visual field of the animal may result from either the motion of the object relative to the animal, or else from the motion of the animal itself in the environment. To determine whether the response of SC-dependent cortical visual areas to dots differs between these two types of visual motion, head-fixed mice placed on a treadmill were presented with a dot in each hemifield whose motion was “coupled” to the running speed of the animal (see STAR Methods). In each trial, the dots moved horizontally in a naso-temporal direction across 40° of visual space (Figure 5A; see STAR Methods). After 1 week, during which mice were familiarized with this environment, we imaged the left hemisphere of the visual cortex while alternating between coupled and “uncoupled” stimulus presentations.^26^ During the uncoupled stimulus presentation, we replayed the stimulus of the preceding coupled stimulus presentation, now, however, uncoupled from the animal’s running speed. Uncoupled trials were sorted according to their “visuo-motor divergence,” which was defined as the difference between the replayed stimulus speed and the animal’s actual running speed, averaged over the course of the stimulus presentation (see STAR Methods). Thus, a positive divergence value indicates that the animal was running slower during stimulus replay than during the preceding coupled stimulus presentation. Accordingly, negative divergence indicates that the animal was running faster than during the preceding coupled stimulus presentation. Strikingly, responses of POR and LI were stronger to stimuli with positive visuo-motor divergence compared with visually identical stimuli coupled to the animal’s running speed (Figures 5B–5E). That is, the visual response was larger when the dots moved faster than the running speed of the animal (Figure 5F, positive visuomotor divergence. All statistics given as mean of peak normalized ΔF/F, uncoupled vs. coupled responses; POR: 0.9922 vs. 0.5812; N = 4 mice; p < 0.05. LI: 0.7234 vs. 0.3019; N = 4 mice; p < 0.05). This was not due to a running speed dependent suppression of the visual response. Indeed, in the coupled condition, visual areas trended toward larger responses when the animal ran faster (Figure S7), consistent with the increased gain of visual responses with running speed.^27^ In marked contrast, responses in V1 were similar between the coupled and uncoupled condition regardless of the sign and magnitude of the visuo-motor divergence (Figure 5D–5F). This finding was not explained by confounding eye movements, as no statistical difference in the distribution of eye positions was observed between coupled and uncoupled stimulus presentations in trials with positive visuo-motor divergence (mean ± standard deviation (SD): azimuth offset: 0.14 ± 4.7°. Elevation offset: −0.13 ± 1.2° ; N = 4 mice, n = 220 trials; NS). These data thus demonstrate that, in contrast to more medial areas, the lateral, SC-dependent areas POR and LI respond preferentially to visual stimuli with positive visuo-motor divergence.

**Figure 6:**
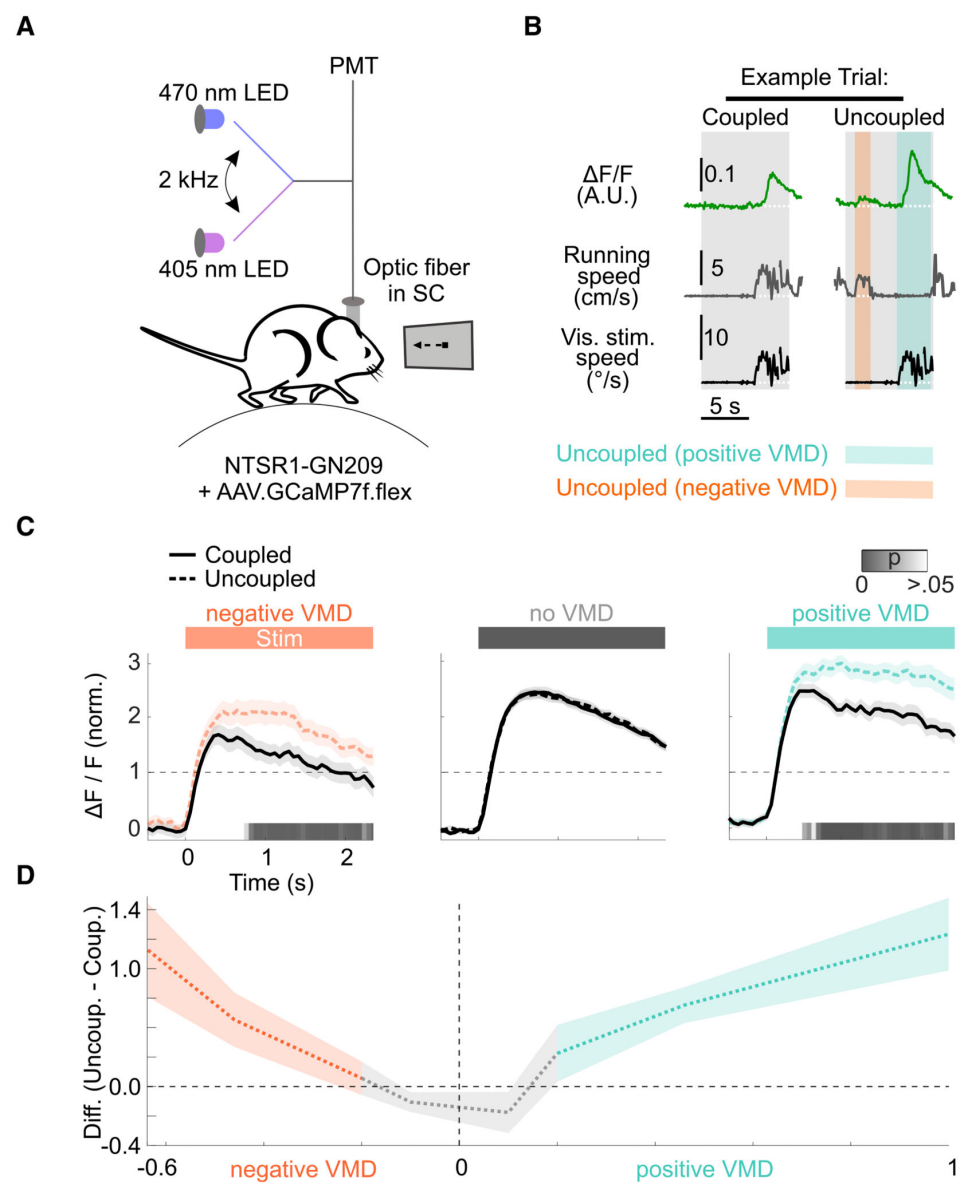
NTSR1-GN209-Cre-tagged cells in SC distinguish between self and externally generated motion of visual stimuli (A) Schematic of experimental configuration for fiber photometry in SC in a mouse running on a treadmill. Two excitation wavelengths at 470 nm (blue; excitation wavelength) and 405 nm (violet; isosbestic hemodynamic control channel) are alternated while imaging visual responses of NTSR1-GN209-Cre-tagged neurons conditionally expressing GCaMP7f. The animal is presented with a dot which progresses along a naso-temporal trajectory either at a speed commensurate to the running speed of the mouse (coupled), or, on alternate trials, with a dot that replays the previous coupled stimulus irrespective of the running speed of the mouse (uncoupled). (B) Example trial illustrating the calcium response (ΔF/F), running speed and the moving dot speed for a pair of coupled and uncoupled stimulus presentations. Negative visuo-motor divergence (VMD; aqua) and positive visuo-motor divergence (orange) events are highlighted during the uncoupled stimulus presentation of the visual stimulus. The horizontal dotted lines indicate baseline for ΔF/F and 0 for running and stimulus speed. A positive visuo-motor divergence event elicits a larger calcium response compared with an identical yet self-generated visual stimulus event. A negative visuo-motor divergence event elicits a response even without movement of the visual stimulus. A.U.:Arbitrary units. (C) Average calcium response in NTSR1-GN209-Cre-tagged neurons in SC for coupled (black solid line) versus uncoupled trials (dashed line). Data plotted as normalized ΔF/F for negative visuo-motor divergence (orange), positive visuo-motor divergence (aqua) and trials with little visuo-motor divergence (light gray). Time zero indicates the onset of the visual stimulus on the monitor. Inset: heatmap of p values for the comparison of the average response on coupled and uncoupled trials, with all p values < 0.05 indicated in gray. N = 3 mice. For positive, negative, and trials with little visuo-motor divergence, n = 194, 114, and 387 trials respectively. p values are calculated with Benjamini-Hochberg method to control false-discovery rate from multiple comparisons. Data plotted as mean ± SEM (shaded area). (D) Difference in calcium response in NTSR1-GN209-Cre-tagged neuronss between pairs of coupled and uncoupled stimulus presentations. Zero on the x axis indicates that the dot moved at the same speed during the coupled and uncoupled stimulus presentation, whereas positive and negative values represent positive visuo-motor divergence (aqua) and negative (orange) visuo-motor divergence trials, respectively. The calcium response in SC is stronger for positive visuo-motor divergence and negative visuo-motor divergence during uncoupled replays compared with stimulus presentations coupled to the running speed of the mouse. Data plotted as mean ± SEM (shaded area).

Is the ability to distinguish between self and externally generated motion already present in the SC, the source of visually evoked activity in these lateral visual areas? To address this possibility, we monitored the activity of NTSR1-GN209-Cre-tagged cells in the SC using fiber photometry. To this end, we conditionally expressed GCaMP7f via local AAV injection and trained mice in the visuo-motor coupling behavior described above. Our data show that, similar to POR and LI, NTSR1-GN209-Cre-tagged cells in the SC respond stronger to positive visuo-motor divergence compared with visually identical stimuli coupled to the animal’s running speed (Figures 6A–6C) (uncoupled mean = 2.222; coupled mean = 1.635; N = 3 mice; p < 0.05). In contrast to LI and POR, however, the SC response for negative visuo-motor divergence was also enhanced compared with visually identical coupled stimuli (Figure 6D; uncoupled mean = 1.626; coupled mean = 1.234; N = 3 mice; p < 0.01). These data indicate that NTSR1-GN209+ cells in the SC differentially respond to visual stimuli depending on whether they are self or externally generated and suggest that this distinction may be in part inherited by lateral HVAs.

## Discussion

This study reveals the presence of a system of lateral HVAs that depends on the SC and that, in contrast to V1, responds preferentially to stimuli with positive visuo-motor divergence. The SC-dependency of each of these lateral HVAs matches the gradient of axonal arborizations arriving in visual cortex from SC through the pulvinar. A genetically defined neuronal population in SC that projects to the pulvinar represents the main conduit of visual information from SC to these lateral HVAs.

We cannot exclude the possibility that HVAs that are medial to V1 and whose visual fields are biased toward lower elevations (e.g., the rostrolateral area; RL^28^) could also have been affected by SC silencing had our visual stimuli been targeted toward the lower extreme of the visual field and our perturbations specifically targeted to the corresponding areas of SC. However, anatomical evidence^10^ indicates that the SC-recipient region of the pulvinar projects preferentially to lateral HVAs, which have visual fields biased toward higher elevations. These observations are consistent with our characterization of a preferential functional impact of SC onto lateral HVAs.

Although pharmacological, optogenetic or surgical approaches have proven to be a potent technique for the identification of SC-dependent visual responses in the cortex of primates,^11,12^ cats,^14^ and rodents,^9,15,16^ they do not allow us to identify which branch, the tecto-pulvinar or the tecto-geniculate^1,2^ is responsible for those responses. Indeed, SC-dependent responses in visual cortex may not only rely on the now well-established tecto-pulvino-cortical route^4,5,9,10^ but also on the disynaptic tecto-geniculo-cortical route.^2,16,29,30^ It is therefore remarkable that expression of TeLC in NTSR1-GN209-Cre-tagged cells of the SC, a perturbation that, among the two main tecto-thalamic pathways,^1^ targets the tecto-pulvinar pathway with great specificity,^18^ is sufficient to reduce responses to moving dots with a similar effect size to TTX injection in the SC. The elimination of the response to moving stimuli with TeLC in SC-dependent cortices is consistent with the functional characteristics of the cells labeled by the NTSR1-GN209 line in the SC, namely the widefield neurons.^18^ These functionally and morphologically defined retinorecipient cells^31^ have large receptive fields and respond specifically to small, high contrast stimuli moving slowly in the visual field along a continuous trajectory.^18^ It thus appears that the functional properties of widefield cells are transmitted to SC-dependent areas of the visual cortex. In contrast, TeLC expression in NTSR1-GN209-Cre-tagged cells had a smaller effect on cortical visual responses to patches of drifting grating as compared with TTX injection in the SC, and this effect was also more restricted to lateral HVAs. There are a few possibilities that could account for the differences observed between these two perturbations. First, the response to patches could be conveyed to cortex via the tecto-geniculate pathway^16,30^ and from there to lateral HVAs.^32^ Alternatively, the remaining responses to patches of drifting gratings could be explained by the difference in the timescales of the perturbations and the ensuing plasticity of alternate routes for visual information. TTX is acute, lasting no more than a few hours, while TeLC is chronically expressed with a 2-week gap between AAV injection and imaging. Thus, the absence of drive from the tecto-thalamic pathway might have promoted, over time, the strengthening of the V1 afferents onto lateral HVAs.

Most moving stimuli in the visual field are generated by the animal as it moves through the environment. We show that SC-dependent cortices distinguish between self and externally generated motion of visual stimuli. We familiarized animals to conditions in which their running speed on a treadmill was coupled to the horizontal motion of a visual stimulus in the opposite direction, a situation in which the motion of the stimulus is entirely generated by the running of the animal, and thus of zero visuo-motor divergence. Following the familiarization period, we compared the visual responses between stimuli whose motion was coupled to the running speed of the animal and identical stimuli moving at the same speed across the visual field that were, however, uncoupled from the animal’s running speed. SC-dependent cortices responded more strongly to the stimulus with a positive visuo-motor divergence, i.e., to the visual stimulus that moves faster than the running speed of the animal. The same was true for NTSR1-GN209-Cre-tagged cells in the SC, suggesting that this property may be inherited by lateral cortical areas directly from the SC. This is consistent with the role of the SC as a detector of visual cues related to threat response^33,34^ and predation.^35,36^ SC-dependent cortices may play a role in the contextual modulation of these behaviors, perhaps representing the valence of the stimuli to guide an appropriate response as has previously been suggested for POR.^37,38^ The preference for positive visuo-motor divergence in SC-dependent cortices also strikes an interesting contrast with previous work describing responses in axonal boutons from pulvinar that terminate in the V1, which instead prefer negative visuo-motor divergence.^39^ This could imply the existence of parallel channels out of pulvinar that segregate different types of discrepancies between actual and expected visual motion and relay them to different visual areas. Consistent with this possibility, we find that NTSR1-GN209-Cre-tagged cells also show enhanced responses to negative visuo-motor divergence.

Cortical visual processing in primates is often described as bisecting between two pathways that extract different types of information from the visual scene: the dorsal stream and the ventral stream.^40^ The extent to which this framework can be applied to rodent vision is the topic of ongoing discussion. In the mouse, connectivity profiling techniques have revealed distinct groupings of HVAs across the lateromedial axis, with a putative ventral stream represented by lateral HVAs and a putative dorsal stream by more medial areas.^41^ This organization is consistent with work demonstrating the role of POR in attributing reward valence to visual stimuli.^37,38^ However, recent considerations have also provided evidence for the opposite assignment, arguing that in non-primate mammals, visual cortical areas that receive tecto-pulvinar input instead make up what is functionally considered the dorsal stream in primates.^42^ This argument is founded on the idea that in primates, dorsal stream area MT, an area that is exquisitely sensitive to moving stimuli, emerged from lateral (temporal) cortex and that its tecto-pulvinar inputs^4,5,11,12^ were evolutionarily outcompeted by the geniculo-striate pathway.^43^ Thus, in species with no area MT, the dorsal/ventral dichotomy may in fact be less suitable than a geniculo-striate/tecto-thalamic framework.^42^

Lateral HVAs are considered the gateway of visual information to the hippocampus.^44^ Afferent projections from lateral visual areas to the entorhinal cortex and hippocampal formation^44,45^ indicate a possible route through which the tecto-pulvinar pathway could influence representations of space in the mouse brain. Determining the extent of this influence is an area of significant future interest, one made more tractable by the method reported here to specifically silence the output to pulvinar from the SC.

## Star⋆Methods

### Key Resources Table

**Table:** 

REAGENT or RESOURCE	SOURCE	IDENTIFIER
Bacterial and virus strains		
AAV1 .CAG.TdTomato.WPRE.SV40	Univ. of Pennsylvania Viral Vector Core	Penn Vector Core ID: p3365
AAV1 .hSyn.Cre.WPRE.hGH	Univ. of Pennsylvania Viral Vector Core	Penn Vector Core ID: p2676
AAV8.CAG.Flex.GFP	Univ. of North Carolina Viral Vector Core	Addgene Catalog # 51502-AAV8
AAV1 .CBA.Flex.TeLC-GFP	Janelia Viral Tools	N/A
AAV9.Ef1α.DIO.ChR2(H134-R).eYFP.WPRE.hGH	Univ. of Pennsylvania Viral Vector Core	Addgene catalog #: 20298-AAV9
AAV2.1.CAG.Flex.jGCaMP7f.WPRE	Dana et al.^46^	Addgene Catalog #: 104496-AAV1
Chemicals, peptides, and recombinant proteins		
Tetrodotoxin	Abcam	Catalog #: ab120055
Cholera Toxin-B/Alexa 488 conjugate	Thermo Fisher	Catalog #: C34775
Experimental models: Organisms/strains		
NTSR1–GN209–Cre	GENSAT	RRID:MMRRC_030780-UCD
Ai14b	Jackson Laboratory	RRID:IMSR_JAX:007914
CamKII-tta x tetO-Gcamp6s	Jackson Laboratory	RRID:IMSR_JAX:007004, RRID:IMSR_JAX:024107
Gad2-Cre	Jackson Laboratory	RRID:IMSR_JAX:010802
Software and algorithms		
Psychophysics toolbox	Brainard et al.^47^	http://psychtoolbox.org
NeuroGit	Caudill^48^	https://github.com/mscaudill/neuroGit
Widefieldlmager	Couto et al.^49^	https://github.com/musall/WidefieldImager
Photometry Analysis	This manuscript	https://doi.org/10.5281/zenodo.8475 https://zenodo.org/badge/latestdoi/623266997
Electrophysiology Acquisition	Hill et al.^50^	https://github.com/danamics/UMS2K

### Resource Availability

#### Lead contact

Further information and requests for resources and reagents should be directed to and will be fulfilled by the lead contact, Dr. Massimo Scanziani (massimo@ucsf.edu).

#### Materials availability

This study did not generate new unique reagents.

### Experimental Model and Subject Details

#### Animals

All experiments were carried out according to regulation from the Institutional Animal Care and Use Committee of the University of California, San Francisco. Data were collected from male or female mice from a C57B6 background. Transgenic lines include NTSR1–GN209–Cre (RRID:MMRRC_030780-UCD), Ai14b (RRID:IMSR_JAX:007914), CamKII-tta x tetO-Gcamp6s (RRID:IMSR_JAX:007004, RRID:IMSR_JAX:024107), and Gad2-Cre (RRID:IMSR_JAX:010802). Mice used for the experiments were at least two months old. Mice were housed on a reverse light cycle (12/12 hours) and experiments were carried out during the dark cycle.

### Method Details

#### Surgeries

Mice were anesthetized with 2% isoflurane and the surgical site was shaved. Animals were placed in a stereotax (Kopf) and a protective petroleum ophthalmic ointment was applied to the eyes. The surgical site was sterilized with saline solution, alcohol, and povidone-iodine (betadine). For pre-operative analgesia, topical lidocaine cream (2%; Akorn Pharmaceutical) was applied to the surgical site and buprenorphine (.1 mg/kg) and carprofen (5 mg/kg) were injected subcutaneously. Subcutaneous analgesics were readministered 6, 12, and 24 hours following surgery.

##### Headstage Surgery

To prepare the mouse for imaging, the skull over visual cortex and surrounding regions (approx. -1.5 to -4.3 mm posterior from bregma, 0-4.1 mm from midline) was thinned with a microburr (Gesswein) mounted in a dental drill (Foredom). Clear dental cement (Triad, Pearson Dental) was evenly distributed across the thinned skull and cured with blue light. A custom triple-threaded headstage was attached to the anterior unthinned portion of the skull (~ 0 to -1.5 mm posterior from bregma along the midline) with transparent cyanoacrylate glue and dental cement (Ortho-Jet, Lang Dental). At least one week was allowed for recovery before imaging

#### Stereotaxic Injections

A ~50 μm craniotomy was drilled with a microburr (Gesswein) mounted in a dental drill (Foredom). Viral solutions were withdrawn into machine-pulled and beveled glass pipettes (tip diameter: 20-40 μm) and then injected with a micropump (UMP-3, WPI) at 40-50 nl/minute at volumes and stereotactic coordinates described below. At the end of the injection, the pipette was left at the injection site for 20 minutes before being slowly retracted and the incision sutured.

##### TTX Injection in SC

We performed craniotomies as described above (see stereotaxic injections) over the SC of mice anesthetized with isoflurane. After allowing 4 hours of recovery from anesthesia, during which time the craniotomy was filled with a surgical silicone adhesive (Kwik-Sil) to maintain sterility, we head fixed the animal and used a glass beveled pipette (tip 20-40 um in diameter) and infusion pump (NanoJet Stereotaxic Syringe pump, Chemyx) to inject 250 nl of TTX at a concentration of 30 μM diluted in PBS, at a speed of 30 nl/min. The injection of TX targeted the stratum opticum of the SC (+0.2 mm anterior from lambda, 0.7 mm from midline, 1.2 mm below pial surface). The pipette was front-filled with a small volume of mineral oil to prevent the drug from leaking into superficial tissue as it was lowered and coated in DiI lipophilic dye (Life Technologies) so that the injection site could be confirmed post hoc. 20 minutes after the end of the injection, visual stimuli were presented and the ipsilateral visual cortex was imaged as described above. In three out of the five mice included in Figure 1, we performed histology to confirm the location of the injection.

##### TeLC Injection in SC

We injected 400 nl of AAV.2.1.flex.TeLC.GFP diluted 1:1 (~5*10^12^ copies/ml) in PBS at a stereotactic coordinate corresponding to the stratum opticum of the left SC (+0.2 mm anterior from lambda, 0.7 mm from midline, 1.2 mm below pial surface) using the viral injection protocol described above. The right SC was left intact. Experiments were performed at least 2 weeks after the injection. Mice were perfused within one week of the experiment so that injection location and viral expression could be confirmed histologically.

##### GCaMP7f Injection in SC

We injected 400 nl of AAV.2.1.flex.GCaMP7f diluted 1:10 (~1*10^12^ copies/ml) in PBS at stereotactic coordinates corresponding to the stratum opticum of the left SC (+0.2 mm anterior from lambda, 0.7 mm from midline, 1.2 mm below pial surface) using the viral injection protocol described above. After the viral injection, a fiber optic cannula, constructed from ceramic ferrule (CFLC440-10, Thorlabs) with 400 um optic fiber cores (0.39 NA, FT400UMT, Thorlabs), was implanted at the respective stereotactic coordinates though positioned 0.2 mm more superficially (1mm depth), and permanently fixed in place with dental cement (Metabond). Mice were habituated to running for several days before the onset of the Calcium imaging experiment. Mice perfused after the full set of experimental sessions, no more than 2 months after the injection, and the injection location and viral expression was confirmed histologically.

##### Anterograde Tracing from SC

We injected NTSR1-GN209-Cre mice with a single 50 nl bolus of a 1:1 mixture of AAV8.CAG.Flex.GFP and AAV1.CAG.TdTomato.WPRE.SV40, each diluted to a 2*10^12 copies/ml titer, in the superficial and intermediate layers of the SC (0.2 mm anterior and 0.7 mm lateral from lambda at 1.2 mm from the pial surface). Mice were perfused after three weeks and sample tissue was mounted for imaging, as described below.

##### V1 Injections for Optogenetic Silencing and Identification of Area LM

We injected Gad2-Cre mice with a single 50 nl bolus of a 1:1 mixture of AAV1.CAG.TdTomato and AAV9.Ef1α.DIO.ChR2(H134-R).eYFP.WPRE.hGh, each diluted to a titer of 2*10^12^ copies/ml, in V1 of the left hemisphere (0.5 mm rostral from lambdoid suture, 2.5 mm lateral from lambda, and 0.7 mm rising to 0.4 mm below the pial surface). After two weeks, the skull was thinned above the visual cortex with a microburr (Gesswein) mounted in a dental drill (Foredom). Under fluorescent illumination (554 nm), the semi-transparent thinned skull revealed the injection site in V1 along with clusters of tdTomato fluorescence marking the corresponding retinotopic location in several higher visual cortices surrounding V1, including LM. LM was carefully inspected for any off-target contamination of ChR2-EYFP to avoid direct optogenetic silencing of LM (Figure S6D).

##### SC Injections for Optogenetic Silencing

We injected Gad2-Cre mice with a single 50 nl bolus of a 1:1 mixture of AAV9.Ef1α.DIO.ChR2(H134-R).eYFP.WPRE.hGh diluted to a titer of 1*10^12^ copies/ml, in SC of the left hemisphere (0.2 mm anterior and 0.7 mm lateral from lambda at 1.2 mm from the pial surface). Mice were perfused after three weeks and sample tissue was mounted for histology, as described below.

##### Trans-Synaptic Anterograde Tracing from SC and Anterograde Tracing from V1

We injected the trans-synaptic anterograde virus AAV1.hSyn.Cre.WPRE.hGH undiluted at a titer of 1.8*10^13^ copies/ml in three separate 70 μl boli in the superior colliculus (0.2 mm anterior and 0.7 mm lateral from lambda at 1.3, 1.2, and 1.1 mm depth) using the protocol described above. One week later, we injected a single bolus of 40 nl of AAV8.CAG.Flex.GFP (diluted to a titer of 10^12^ copies/ml) in the caudal portion of the pulvinar (2.7 mm posterior and 1.7 mm lateral from bregma, 2.6 mm below the pial surface) and a single bolus of 50 nl of AAV1.CAG.TdTomato.WPRE.SV40 (diluted to a titer of 10^12^ copies/ml) in V1 (0.5 mm rostral from lambdoid suture, 2.5 mm lateral from lambda, and 0.7 mm rising to 0.4 mm below the pial surface). Three weeks after the second set of injections, mice were perfused and sample tissue was mounted for confocal imaging, as described below.

##### Retrograde Tracing from Pulvinar

We injected a single bolus of 20 nl of the retrograde tracer Cholera Toxin-B/Alexa 488 conjugate, in the pulvinar (2.7 mm posterior and 1.7 mm lateral from bregma, 2.6 mm below the pial surface) of double-transgenic mice (NTSR1-GN209-Cre x Ai14b). Mice were perfused after one week and sample tissue was mounted for confocal imaging and validation of injection site, as described below.

#### Histology

For all anatomical analyses, mice were transcardially perfused with 10 ml of cold (4°C) phosphate buffered saline (PBS), followed by 10 ml of 4% paraformaldehyde (PFA) in PBS. Brains were extracted and set to fix overnight at 4°C in 4% PFA solution. After post-fixation, brains were cut with a vibratome to 50 μm thickness and mounted on slides with Vectashield mounting medium containing DAPI (Vector Laboratories H1500). Lower magnification images were taken with an Olympus MVX10 MacroView microscope, while higher magnification images (including all images analyzed quantitatively) were taken with a Nikon Ti CSU-W1 inverted spinning disk confocal microscope. In the experiments where we injected AAV.2.1.flex.TeLC.GFP in NTSR1-GN209-Cre mice, we verified the accurate targeting of SC and viral spread through the local expression of GFP. The diameter of the viral spread was quantified based on the Feret diameter with ImageJ for a series of coronal sections spanning SC and averaging across sections.

#### Widefield Calcium Imaging

We head-fixed double transgenic mice (CAMKII-TTA x TetO-GCaMP6s) beneath a widefield microscope (Olympus MVX10, Olympus MV PLAPO 2XC). The field was illuminated with blue light (470 nm, filtered with Olympus OCT-49020MVX NB) from a mercury lamp (Olympus U-RFL-T, OSRAM HBO 100 W/2) at a maximum of 3.2 mW/cm^2^ (about 10 mW total). Light transmitted through the objective lens was filtered (Olympus OCT-49020MVX EGFP) and images captured with a Hamamatsu C11440 camera. To ensure in-focus capture of signal from the entire left hemisphere of cortex (across the full lateromedial extent, and from -1 mm posterior of bregma to the lambdoid suture), the microscope was rotated to 45-degrees relative to the horizontal plane and an aperture diaphragm above the objective lens was nearly closed to maximize depth-of-field. Because this protocol limited the availability of emitted light from the sample, images were captured with a relatively long exposure of 100 ms. In a subset of control experiments, we compared visual responses with and without correction for the hemodynamic signal using a separate microscope (see below; Figure S8). The hemodynamic correction had little impact on response magnitude and time course and no impact on stimulus preference.

##### Hemodynamic Correction

In a subset of experiments (Figure S8), we compared cortical responses to visual stimuli with and without hemodynamic correction. To determine the impact of local intrinsic hemodynamic changes on our recordings, we interleaved frames illuminated at the approximate isosbestic point for GCaMP6s (405 nm) with frames illuminated using blue light (470 nm). Images were acquired at 60 Hz (30 Hz per channel). We used a custom-built microscope^49^ Imaging lens: Nikon AF DC Nikkor 105mm f2; Objective lens: Sony 50mm f/1.4) and captured frames using a PCO edge 5.5 camera. Frames taken with isosbestic illumination were normalized and subtracted from the dynamic calcium signal and compared to uncorrected signal. Codebase for camera control and analysis is available on github^49^ (https://github.com/musall/WidefieldImager).

#### Fiber photometry

Two weeks after injection of GCaMP7f virus (see injection protocol above) and implantation of the ceramic ferrule, mice were habituated to head-fixation and running on a linear treadmill (as described in the *Animal Generated Motion* protocol below). Fiber photometry data were collected using a photoreceiver (New Focus Visible Femtowatt Photoreceiver, Newport) and demodulated and processed with a RZ5P Processor (TDT). The photometry system was adapted to two excitation LEDs at 470 nm (GCaMP channel) and 405 nm (isosbestic hemodynamic control channel). The two channels were simultaneously sampled via a patch cord (400 μm core). The LEDs were controlled by drivers that sinusoidally modulated the excitation at 405/470 nm at 2 kHz. The fiber-optic patch cord was connected to the fiber implant on the animal via a ceramic mating sleeve (Thorlabs). Imaging data was acquired using Synapse software (TDT) and analyzed using custom written Matlab routines (https://zenodo.org/badge/latestdoi/623266997).

#### Visual Stimuli

We presented visual stimuli on a monitor (ViewSonic VX2452MH, 60 hz, 24”, 1920x1080 resolution, 119.8 cd/m2 maximum luminance, 45.2 cd/m^2^ mean background luminance, 0.8 cd/m^2^ minimum luminance, gamma corrected) centered 15 cm in front of the contralateral eye of the animal. The position of the monitor relative to the animal was angled at 30° from the long body axis and visual coordinates were calculated relative to the midline (azimuth) and the horizontal plane through the animal’s eye (altitude). The monitor covered approximately 120 degrees along the azimuth (0° to 120° in azimuth) and 90 degrees in altitude (-35° to 55° in altitude). IR-illuminated eye movements were tracked with a separate camera (DMK 23UM021). Mice were habituated to this setup at least 4 days before experimental data were gathered. Mice were head-fixed within a polycarbonate tube to minimize stress, except where otherwise noted for experiments related to animal generated motion. Stimuli were generated using modified code from the psychophysics toolbox^47^ (http://psychtoolbox.org) and NeuroGit^48^ (https://github.com/mscaudill/neuroGit).

##### Identification of HVAs

To approximate the location of higher visual areas, we presented circular patches of sinusoidal drifting grating (consisting of a circular 10 degrees drifting grating with spatial frequency: 0.05 cycles/degree, temporal frequency: 1.5 Hz, contrast: 100%) at 5 different locations in the shape of a plus sign in front of a mean luminance gray background, with one patch at the center of the plus (0,0 on the monitor; Figure S1) and the others at the end of each arm (± 15 degrees in X or Y). Each patch was offset from the location of the center patch by 15 degrees along either the azimuth or elevation axis. Each patch was presented individually. Patches were randomized and interleaved, appearing for one second per presentation either 30 or 60 times in each location, with 4 seconds allowed between each presentation. Average responses for patches in each position were generated, then binarized with a threshold and overlaid to produce retinotopic maps for each mouse. These maps were used to determine the regions of interest for calcium responses in subsequent experiments. To ensure that visually evoked responses to other stimuli (e.g. dots) could be properly attributed to the delineated areas, those other stimuli were presented within the visual field boundaries mapped by the patches. In addition, we generated visuotopic maps using a spherically-corrected checkerboard visual stimulus drifting across the visual field to delineate the borders between field sign patches^51^ (Figure S2). In brief, we used a moving bar containing a flickering black-and-white checkerboard pattern to stimulate visual cortical areas based on spherical visual coordinates using a planar display.^52^ To generate an altitude or azimuth map, the moving bar was swept across the monitor ten times along the vertical or horizontal axis respectively. Visual field maps were generated based on azimuth and altitude maps using published Python code.

##### Drifting Dots

We presented small (two degrees) black squares in front of a mean luminance gray background moving across a vertical 30-degree trajectory in one second. The dots were presented within the visual field boundaries mapped with the grating patches (see above). Continuous dots were presented either 60 or 120 times each, vertically downwards and vertically upwards, before all responses were averaged together.

##### Drifting Gratings for Extracellular Recording

Circular patches of sinusoidal drifting gratings (consisting of a circular 20-45 degrees drifting grating with spatial frequencies: 0.04-0.05 cycles/degree, temporal frequencies: 1.5-3Hz, contrast: 100%, drifting in 1-4 cardinal directions) were displayed at the center of the receptive field of the V1 recording site (see below). The stimuli were presented for 0.9-1.5s, and preceded and followed by a mean luminance gray screen.

##### Animal-Generated Motion

For experiments described in Figures 5 and 6, animals were head-fixed and trained to run on a cylindrical styrofoam wheel. A linear encoder (US Digital MA3-A10-125-8) was attached to the center of the wheel to relay the running speed of the mouse to a National Instruments Data Acquisition device (NI USB6001). A monitor was placed on each side of the animal. The two monitors had an angle of ~60 degrees relative to each other, converging in front of the animal. A 2-degree black dot appeared on the gray monitor at the nasal end of the visual field boundaries mapped with the patches and moved horizontally in the temporal direction for 40 degrees at a speed that was proportional to the running speed of the animal (up to ~30 deg/s) before disappearing, marking the end of the trial (coupled trials). The dot remained at the same elevation throughout the experiment. Individual trials were separated by a 4s inter-trial interval. After each trial, (500 ms after the disappearance of the dot) a small soymilk reward was administered to the animal through a lickport. Mice were familiarized on this setup 30 - 60 min/day for approximately one week, until they learned to run continuously throughout a 30-minute block (excluding inter-trial intervals). After the familiarization, on the day of the experiment, coupled trials were interleaved with uncoupled trials, i.e. trials that replayed the previous coupled stimulus, now, however, uncoupled to the mouse’s running speed. Both coupled and uncoupled trials were paired with the same soymilk reward and 4-second inter-trial interval. Visually identical coupled and uncoupled trials were analyzed as described below.

#### *In Vivo* Electrophysiology

Extracellular recordings from V1, SC, LVA were performed using the following NeuroNexus silicon probes:

A1x32-Edge-5mm-20-177-A32;

A4x2-tet-5mm-150-200-121-A32;

A1x16-5mm-25-177-A16;

A1x16-5mm-25-177-OA16LP (optrode; optical fiber: 0.66NA, 105μm core, 125μm cladding).

Two small craniotomies (~50 μm in diameter) were drilled over the V1 tdTomato injection site (see stereotaxic injections) and the cluster of tdTomato fluorescence corresponding to the lateral higher visual area of interest, the precise coordinates of which varied depending on the experiment. For experiments in which SC recordings were also taken, a third craniotomy was drilled over the SC injection site (see stereotaxic injections). Multichannel linear probes (Neuronexus) were mounted on micromanipulators (Luigs & Neumann), coated with DiI or DiO dyes (Life Technologies), and placed in V1, the SC, and/or a lateral higher visual area of awake, head-fixed mice.The recording area was protected by a recording chamber built with dental cement (Ortho-Jet powder; Lang Dental) mixed with black paint (iron oxide), and filled with artificial cerebrospinal fluid (ACSF; 140 mM NaCl, 5 mM KCl, 10 mM d-glucose, 10 mM HEPES, 2 mM CaCl2, 2 mM MgSO4, pH 7.4). The recorded signals were amplified and filtered using A-M System head stages (gain 20x) and A-M System amplifiers (Model 3500 and 18 Model 4000, gain 100x, band pass filter: between 0.3Hz or 0.1Hz and 5KHz). Signals were acquired at 25 KHz or 32 KHz using one or two data acquisition boards (NIDAQ PCIe-6259) and spike-sorted through the Matlab-written software package^50^ (https://github.com/danamics/UMS2K).

##### Optogenetic Silencing of V1

We silenced V1 by optogenetically activating local inhibitory interneurons expressing Channelrhodopsin 2 (ChR2; see injection protocol above). This approach has been demonstrated to completely silence V1 responses to visual stimuli across cortical layers.^9,23,53^ During electrophysiological recordings, ChR2-expressing interneurons in V1 were activated through an optical fiber (1mm; Thorlabs) connected to a blue LED (470nm; Thorlabs). The fiber was positioned on the surface of V1, above the tdTomato injection site, and hence in a visuotopically matched location relative to the LM recording site. Additionally, the visuotopic match of V1 and LM recording sites was verified by simultaneously measuring the receptive fields of multiunit activity in each site. The power of the LED (3mW, measured at the fiber tip) was assessed before each experiment through a digital power meter (PM100D, Thorlabs). During the electrophysiological recordings, trials of V1 silencing were interleaved with control trials as patches of drifting grating (see above) were presented. During trials with optogenetic silencing, the LED was activated before and during the presentation of the visual stimulus.

##### Optogenetic Silencing of SC

We silenced SC by local optogenetic activation of glutamic acid decarboxylase 2 locus (*Gad2*) interneurons expressing ChR2 (see injection protocol above) via an optic fiber coupled to a silicon probe connected to a blue laser (Omicron Laserage, LuxX 470 nm; 16 channels NeuroNexus optrode, fiber core diameter: 105μm, power measured at the fiber tip: 1mW). This approach has been demonstrated to completely silence neuronal activity in SC at the recording site.^9^ The visuotopic match of SC, V1, and lateral visual cortical recording sites was verified by simultaneously measuring the receptive fields of multiunit activity at each site and adjusting the position of each electrode as needed. The power of the laser was assessed before each experiment through a digital power meter (PM100D, Thorlabs). During the electrophysiological recordings, trials of SC silencing were interleaved with control trials as patches of drifting grating (see above) were presented. During trials with optogenetic silencing, the laser was activated before and during the presentation of the visual stimulus.

### Quantification and Statistical Analysis

#### Histological Analysis

##### Cortical Depth Profiles of Afferent Projections

For analysis of coronal cortical sections (Figure 4), confocal images were acquired as described above and the position of the imaged plane along the anterior-posterior axis was registered to a plane in the Allen Adult Mouse Brain Reference Atlas using the hippocampus as a landmark. HVAs, labeled by the anterograde projection from V1, were ascribed using the atlas, as well as by referencing the positions of each labeled area relative to one another.^24^ Confocal Z projections (maximum intensity) were taken for each stack, each projection was rotated such that the pia was aligned to the y-axis and the x-axis corresponded to cortical depth. A boundary box was drawn such that the x-axis extended from the pia to the white matter, and the y-axis covered the full extent of the HVA as defined by the TdTomato expression. The raw fluorescence density (for both tdTomato and GFP) at each x-position was calculated as the average fluorescence across the y-axis. Because autofluorescence varies along the depth of each section, a baseline depth profile (for both tdTomato and GFP) was calculated separately for each section by sampling points with no visible labeling along the full depth (x-axis) of the section. This profile was then subtracted from the raw fluorescent density. The fluorescent density was computed as the baseline subtracted raw fluorescent density, normalized by the maximum across all sections in a given mouse (independently for GFP and tdTomato). For areas extending across more than one section of tissue, boxes were drawn across every section and broken into 10 bins. Fluorescence density depth profiles were then constructed by selecting each bin from the section with the greatest average fluorescent density and concatenating the bins into a full profile. The total fluorescence of a HVA was calculated as the sum of pixels across all x-y. The fraction of total fluorescence of a given HVA was the total fluorescence of that HVA normalized by the sum of total fluorescence of all HVAs. Coloration for insets to box plots in Figure 4 was generated by interpolating the average fraction of total fluorescence for each area into an 8-bit RGB color scheme in either the green or red channel.

##### Determining Percent Overlap of Retrograde Tracer with TdTomato

After confocal stacks were acquired as described above, color channels containing signals for Alexa 488 and TdTomato were split for separate analysis. Positive cells were manually identified based on size and shape, and separately labeled using the cell counter plugin for ImageJ. The channels were then merged and counts were taken for double-labeled cells.

#### Measuring Activity in Visual Cortex

Responses to experimental stimuli were either recorded in the same session as the HVA identification protocol, ensuring that areas remained aligned to their maps, or else image registration based on vascularization was used to align areas *post-hoc*. Visual responses were averaged over all trials, from the onset of the visual stimulus until 0.5 s after its offset (1.5 s total). Baseline was collected from one second before the onset of each stimulus, averaged, and used to generate ΔF/F. Moving dots were presented only within approximately one half of the visual space covered by the visual stimuli used to create HVA maps; therefore, rather than computing responses to moving dots over the entire area of a given HVAs, only the top 50% of responding pixels were used for analysis. For intra-hemispheric comparisons, an average image of the control and experimental mean response was used to select the 50% most responsive pixels, ensuring an unbiased selection of pixels for the comparison between different conditions. For inter-hemispheric comparisons, responses in each area were first normalized to the response in V1 of the same hemisphere to account for differences in expression or preparation across hemispheres.

##### Coupled and Uncoupled Trials

Uncoupled trials were sorted in three groups: Trials in which the visual stimulus speed was at least 25% greater than the running speed were labeled as “positive visuo-motor divergence” (approx. 30% of trials); trials in which the difference was less than -25% were labeled as “positive visuo-motor divergence” (approx. 30% of trials), and all other trials were labeled as “matched”. For time courses and difference plots in Figures 5B–5D, responses in each higher visual area were normalized to the mean V1 response across all coupled trials for each mouse over the first 1.7 seconds of the trial. Because, in the coupled condition, the duration of the presence of the visual stimulus on the monitor varies from trial to trial depending on the running speed of the animal, the average time-course of the cortical response contains a progressively smaller number of trials as time advances from the onset of the stimulus. We thus analyzed only the response during the first 1.7 s from stimulus onset; In the “positive visuo-motor divergence” condition, at 1.7 s the stimulus was still present in the following proportions of trials: for mouse 1, 67.5%; for mouse 2, 78.2%; for mouse 3, 87.9%; and for mouse 4, 94.6%. Each mouse contributed equally to the statistics, independently of how many trials it performed. Data in the box plot in Figure 5E were normalized to the maximum response across all areas for each mouse. Data in Figure 6C were normalized to the mean response across all coupled trials for each mouse and then baseline corrected. Data in Figure 6D were first split into bins based on size and type of visuomotor divergence. Trials were then normalized to the mean response across all coupled trials for each mouse. These normalized responses from coupled trials were subtracted from normalized responses from uncoupled trials to calculate the mean difference in normalized response for each bin.

#### In vivo Electrophysiology

Spikes were defined as events crossing a threshold of 4 times the standard deviation of the recorded extracellular signal. Spike wave-forms were sorted and classified using a K-means clustering algorithm as regular-spiking neurons (RS) or fast-spiking putative interneurons (FS), as in Beltramo and Scanziani.^9^ Trial-averaged responses to circular drifting grating patches were measured during the time of the stimulus presentation. The responses were baseline subtracted (baseline for experiments with 0.9 s of stimulus presentation: firing rate averaged over 700 ms before the visual stimulus; baseline for experiments with 1.5 s of stimulus presentation: firing rate averaged over 300 ms before the visual stimulus). Isolated units were classified as responsive if the average firing rate during the presentation of the visual stimulus was larger than 2 standard deviations from the baseline for experiments with 0.9 s of stimulus presentation, or larger than 1 standard deviation from the baseline for experiments with 1.5 s of stimulus presentation. The direction of the drifting grating that evoked the largest response was determined for each isolated unit, and the baseline-subtracted average firing rate during the presentation of the visual stimulus was computed in control conditions and with photostimulation. For each unit, we calculated the Z-score of the firing rate (Z-score FR), as: (average firing rate during stimulus presentation - average firing rate during baseline period) / standard deviation of the firing rate during the baseline period. We calculated the trial averaged peristimulus time histogram (PSTH; 50 ms binning) of the firing activity for each isolated unit. We then generated a summary PSTH by averaging the PSTHs of each isolated unit: each trial averaged PSTH of each individual unit was normalized by its maximum value (i.e. the value of its largest 50 ms temporal bin) and the normalized PSTHs of the individual units were averaged together. The PSTH of each individual unit during optogenetic manipulations was normalized by the maximal value of the PSTH of that same unit under control conditions. When comparing different areas, we quantified the effect of the optogenetic manipulation on the firing activity of each unit as a percent reduction of the visual response: (1 – baseline subtracted average firing rate during illumination / baseline subtracted average response in control conditions) x 100.

#### Statistics

All statistical analyses were performed in MATLAB. Data in boxplots are generated using the notBoxPlot script available on github (https://github.com/raacampbell/notBoxPlot) and presented with a red bar representing the mean and a gray or colored box representing 1.96 standard errors (i.e. a 95% confidence interval). For each experiment, only visual areas that were responsive to visual stimulation under control conditions (i.e. response at least 2 standard deviations above baseline) were included in the analysis. Accordingly, the number of data points may differ between HVAs in some of the box plots. Statistics for HVAs, irrespective of whether they respond to visual stimuli, are as follows: for Figure 3: POR: Mean reduction 80.14%; N = 9 mice; p = 5.371*10^-7^. LI: Mean reduction: 70.78%; N = 9 mice; p = 1.033*10^-5^; P: Mean reduction = -133.8% (including one large outlier; Median reduction = 59.6%); N = 9 mice; p = 0.0249. LM: Mean reduction: 36.82%; N = 9 mice; p = 4.399*10^-5^. AL: Mean reduction: 53.3% (including one large outlier; Median reduction = 13.15%); N = 9 mice; p = 0.9095. P-values in time courses in Figures 5 and 6 are calculated using the Benjamini & Hochberg method to reduce the risk of false discovery from multiple comparisons. Statistics for all other experiments can be found in the figure legends or the main text. P-values in electrophysiology experiments were calculated using the Wilcoxon signed-rank test. All other p-values were calculated from paired t-tests in the case of intra-hemispheric comparisons and unpaired t-tests in the case of inter-hemispheric comparisons, presuming a normal distribution and independence although these properties were not established statistically. P-values marked as NS are >.05. Statistical significance is denoted as *p<.05, **p<.01, ***p<.001. Sample sizes used are typical of those employed in the field. Experiments and analyses were unblinded.

## Supplementary Material

Supplementary Materials

## Data Availability

All data reported in this study will be made available by the lead contact (massimo@ucsf.edu) upon request. All original code has been deposited on Zenodo and is publicly available as of the date of publication. DOIs are provided in the key resources table. Any additional information required to reanalyze the data reported in this work paper is available from the lead contact upon request.

## Highlights

- Visual responses in several lateral cortical areas depend on the superior colliculus (SC)
- A genetically defined SC neuron type drives these responses via the pulvinar
- SC impact on these responses decreases along a lateromedial gradient across the cortex
- SC-dependent visual areas discriminate self from externally generated visual motion

## In brief

Brenner et al. report that several mouse lateral visual cortical areas depend on the superior colliculus (SC) for visually evoked activity. Genetically defined pulvinar-projecting SC neurons drive these areas in a topographically organized manner along a lateromedial gradient. SC-dependent visual cortices distinguish between self and externally generated motion.
