# Supplementary material for: A genetically defined tecto-thalamic pathway drives a system of superior-colliculus-dependent visual cortices
Source: Neuron. Author manuscript; Available in PMC 2026 Jun 2. (PMC10524301; doi:10.1016/j.neuron.2023.04.022)
Supplement: Supplementary Materials [file EMS211687-suppement-Supplementary_Materials.pdf]

Neuron, Volume *111*

Supplemental information

A genetically defined tecto-thalamic pathway drives a system of superior-colliculus-dependent visual cortices

Joshua M. Brenner, Riccardo Beltramo, Charles R. Gerfen, Sarah Ruediger, and Massimo Scanziani

Supplementary Figures and Figure Legends

Figure S1

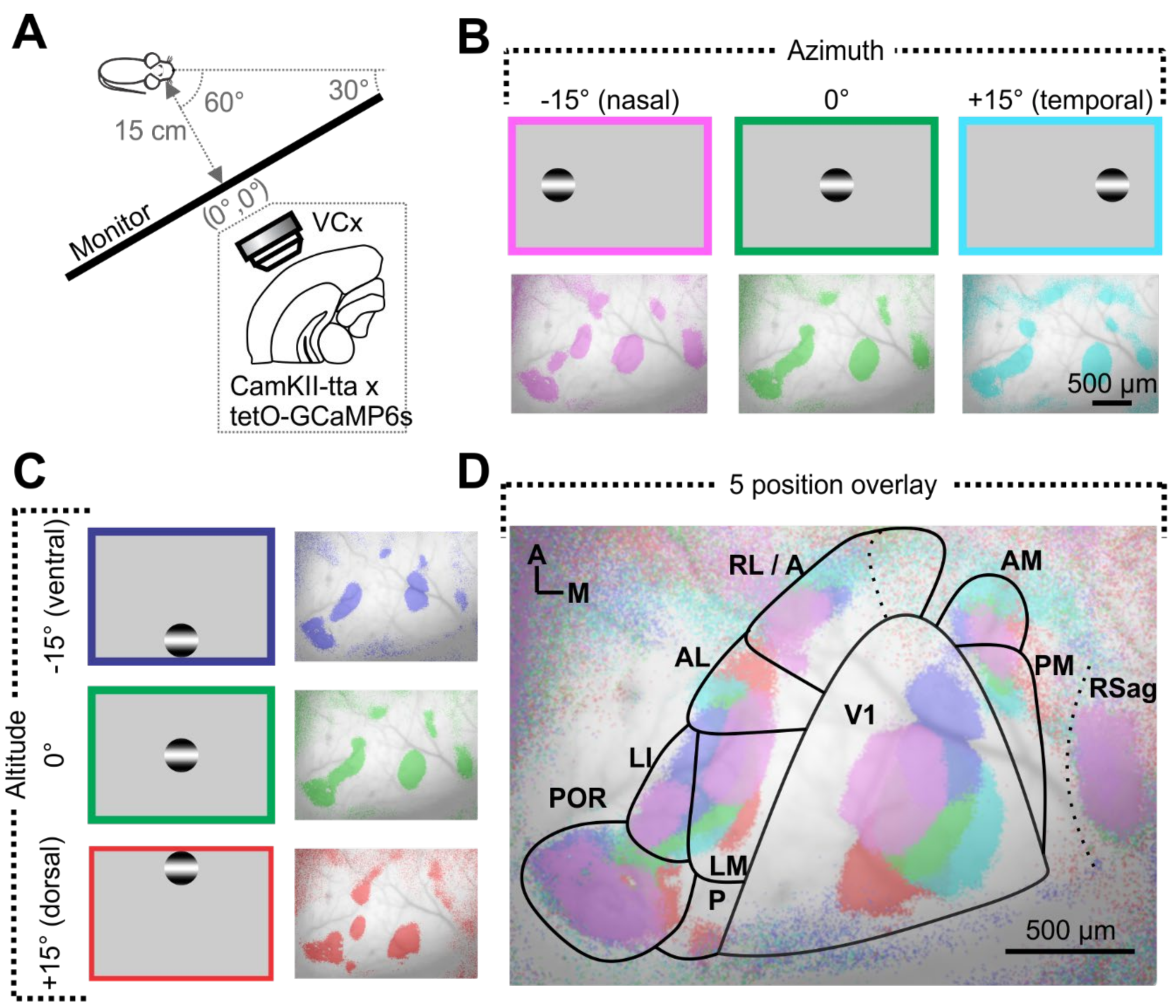

Figure S1. Mapping Visual Cortical Areas, Related to Figure 1.

(**A**) Schematics of experimental configuration for wide-field calcium imaging of visual cortex (VCx). Mice express GCaMP6s driven by the CamKII promoter via a tet-on system.

(**B**) Top row: Patches of drifting gratings (10 degree diameter; 0.05 cycles/degree; 1.5 Hz; 1 s long presentation; 4 s interstimulus interval) are presented at three positions along the azimuth separated by 15 degrees each. Bottom row; Example calcium response to the above stimuli. For mapping visual areas, calcium transients, averaged over 60 trials, were binarized with a threshold (see methods).

(**C**) Same as (B) but with stimuli staggered along elevation.

(**D**) Overlay of all responses showing retinotopy of V1 with HVAs with their estimated boundaries (POR: Postrhinal Cortex; LI: Laterointermediate Area; P: Posterior Area; LM: Lateromedial Area; AL: Anterolateral Area; AM: Anteromedial Area; PM: Posteromedial Area; RL/A: Rostrolateral Area; RSag: Agranular Retrosplenial Cortex; V1: Primary Visual Cortex). Orientation: A: anterior; M: medial.

Figure S2

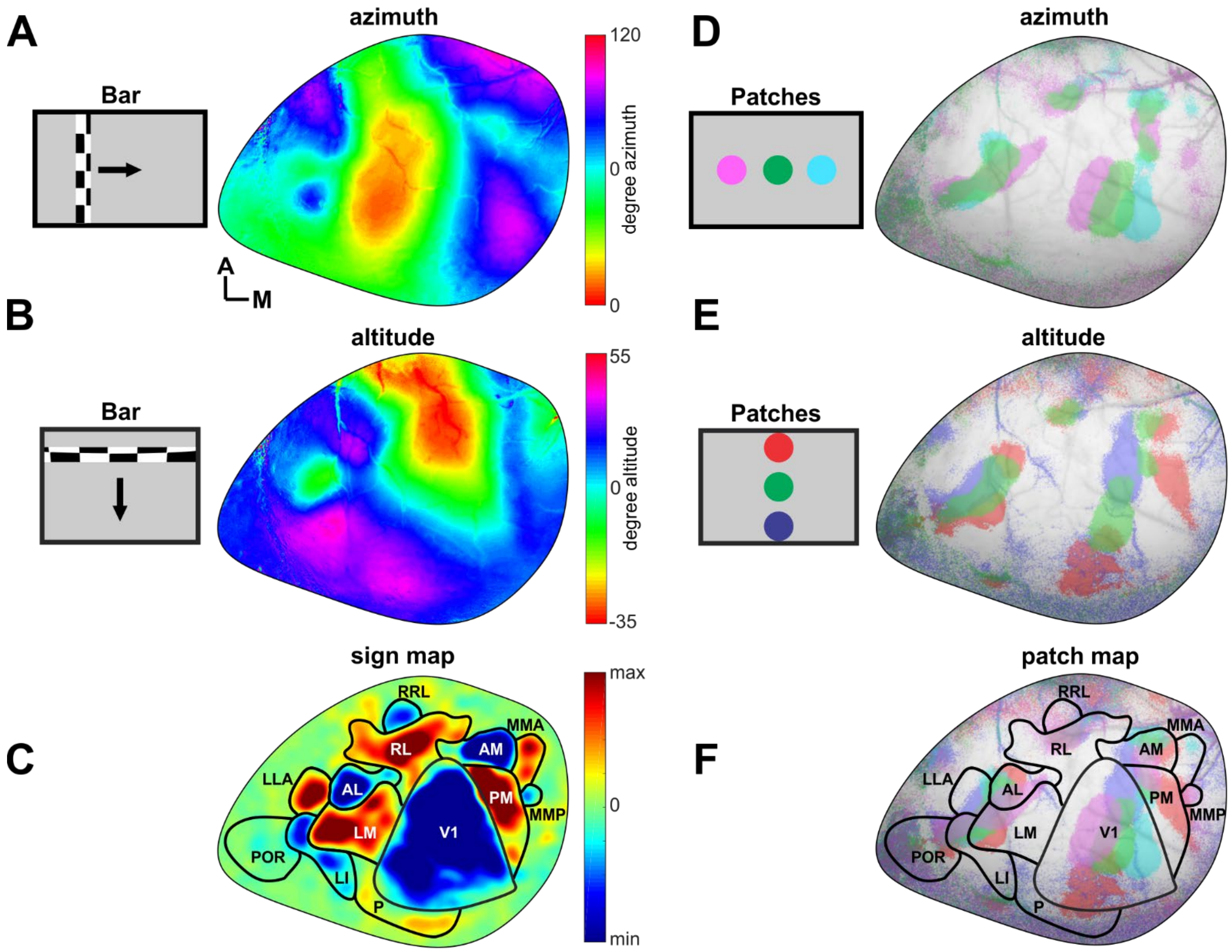
A
azimuth
Bar
120
0
degree azimuth
A
M
B
altitude
Bar
55
0
-35
degree altitude
C
sign map
max
0
min
RRL
MMA
RL
AM
LLA
AL
PM
MMP
LM
V1
POR
LI
P
D
azimuth
Patches
E
altitude
Patches
F
patch map
RRL
MMA
RL
AM
LLA
AL
PM
MMP
LM
V1
POR
LI
P

Figure S2. Comparison of Retinotopic Maps Based on Bar or Patch Stimulus Patterns, Related to STAR Methods: *Identification of HVAs*

(**A**) Visuotopic mapping data based on GCaMP6 fluorescence from an awake, passive mouse presented with a drifting bar containing a flickering checkerboard on a gray screen (see methods) placed on the right visual hemifield. The bar drifted from left to right (nasal to temporal; arrow) on the screen to generate an azimuth map of the visual field. The fluorescence image is based on the response to 10 repetitions of the visual stimulus Orientation: A: anterior; M: medial.

(**B**) As in (A), but with the bar moving from top to bottom to generate an altitude map of the visual field. The image is based on 10 repetitions of the visual stimulus.

(**C**) Field sign map based on the altitude and azimuth maps in (A) and (B). Black borders highlight the boundaries between visual areas. (LLA: Lateral of Anterolateral Area; RRL: Rostral of Rostrolateral Area; MMA: Medial of Anteromedial Area; MMP: Medial of Posteromedial Area; All other abbreviations as in Fig. S1D).

(**D**) Same animal and imaging site as (A-C). Retinotopic mapping data based on patches of drifting gratings presented at three positions along the azimuth (magenta: 15° nasal; green: center; cyan: 15° temporal) as in Figure S1.

(**E**) Same as (D), but patches are presented along the altitude (blue: 15° inferior; green: center; red: 15° superior).

(**F**) Combination of the maps in (D) and (E) overlaid on the sign-map delineated boundaries from (C). Note that the areas identified with the patches are contained within the boundaries delineated by the sign-map.

Figure S3

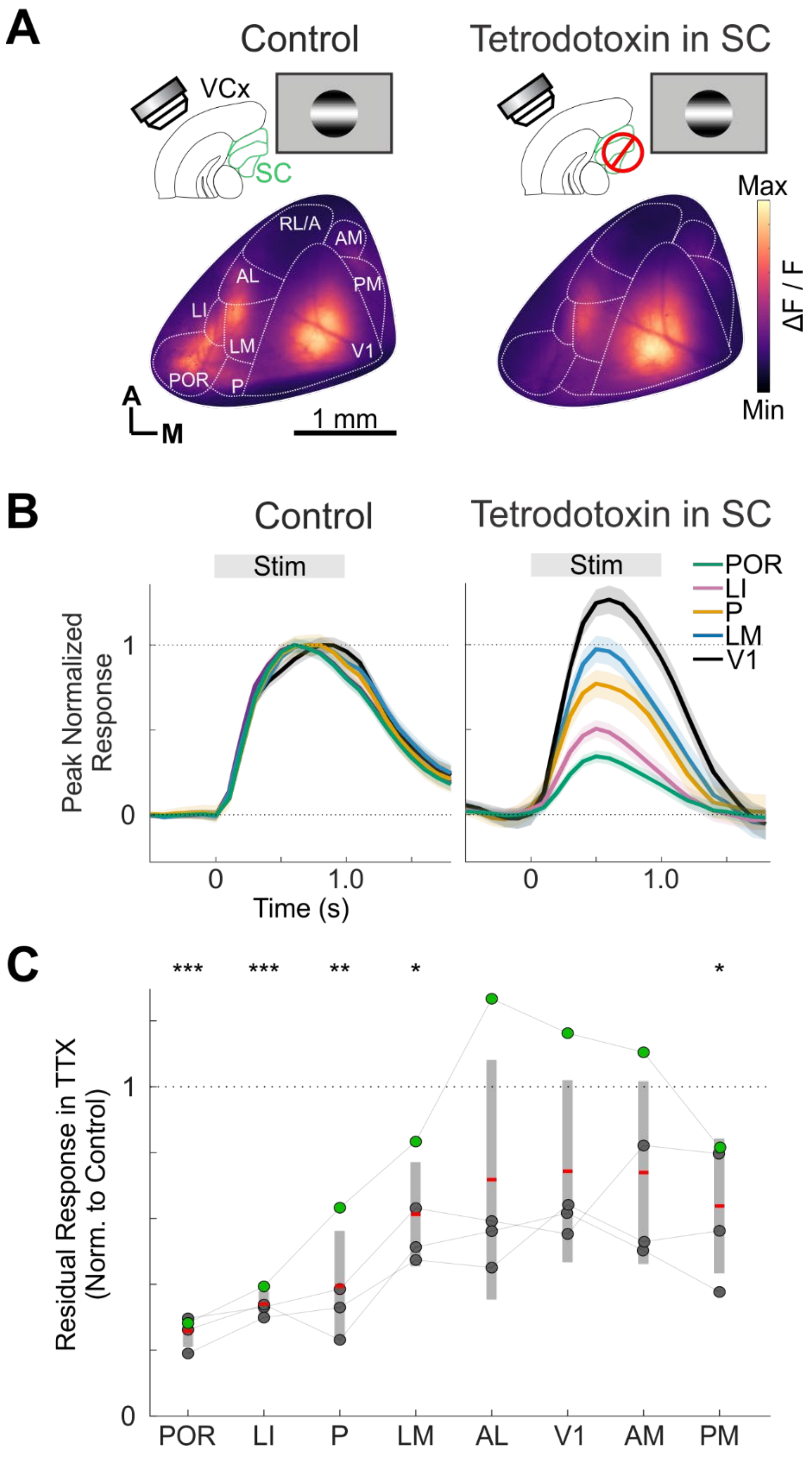
A
Control
Tetrodotoxin in SC
VCx
SC
RL/A
AM
AL
PM
LI
LM
V1
POR
P
A
M
1 mm
Max
ΔF / F
Min
B
Control
Tetrodotoxin in SC
Stim
Stim
POR
LI
P
LM
V1
Peak Normalized Response
1
0
0
1.0
0
1.0
Time (s)
C
*** *** ** * *
Residual Response in TTX (Norm. to Control)
1
0
POR
LI
P
LM
AL
V1
AM
PM

Figure S3. Injection of TTX in SC Reduces Responses to Drifting Grating in Lateral Visual Areas, Related to Figure 1.

(**A**) Example ΔF/F (5 position, 300 trial average) across visual areas before (left) and after (right) TTX injection in SC. Cortical visual areas delineated as shown in Figure S1. Orientation: A: anterior; M: medial.

(**B**) Time courses of calcium response to visual stimulus from the example experiment above. Traces are normalized to peak response in each area under control condition. Shading: SEM

(**C**) Summary data for 4 mice. Y axis plots remaining visual response after TTX injection in SC, normalized by the control response for each area. Gray bar shows 95% confidence interval above and below mean (red line). Gray lines between dots link areas from the same animal. P values are calculated with paired t-test. Green dots indicate the data used in the example in (A) and (B). (Significant reduction: POR: 74.2% reduction; N = 4 mice; $p < 10^{-3}$. LI: 66.0% reduction; N = 4 mice; $p < 10^{-3}$. P: 60.5% reduction; N = 4 mice; $p < 0.01$. LM: 38.7% reduction; N = 4 mice; $p < 0.05$. PM: 36.2% reduction; N = 4 mice; $p < .05$. Not significantly reduced: AL: 28.2% reduced; N = 4 mice; NS. V1: 25.7% reduced; N = 4 mice; NS. AM: 26.1% reduced; N = 4 mice; NS).

Figure S4

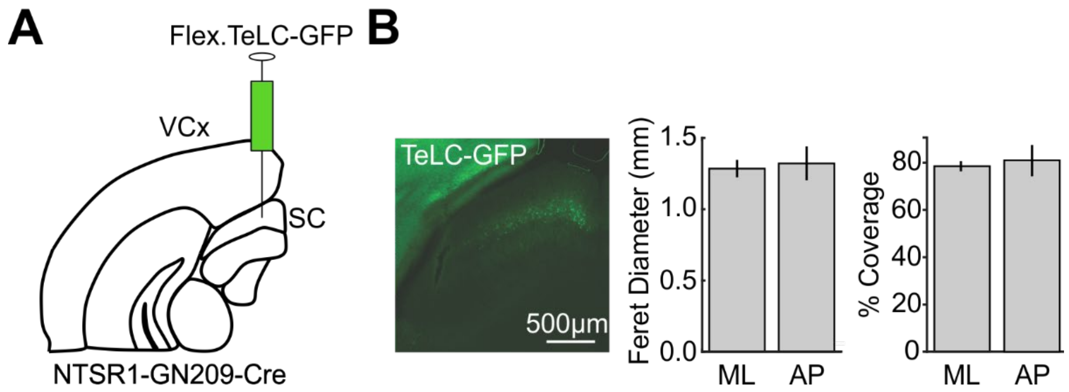

Figure S4. Histological Analysis of TeLC Spread in SC, Related to Figure 3.
(**A**) Schematic of viral injection to conditionally express TeLC in NTSR1-GN209-Cre tagged neurons in SC.
(**B**) Left: Coronal section from an experimental mouse included in Figure 3 illustrating the viral spread of TeLC-GFP. Middle: Diameter of the viral spread along the anterior-posterior and medio-lateral axis based on the Feret diameter. Right. Spatial coverage of the viral spread along the anterior-posterior and medio-lateral axis. Data plotted as mean ± SEM, from 6 mice. ML: mediolateral; AP: anteroposterior. These data indicate nearly full coverage of the SC with TeLC expression.

Figure S5

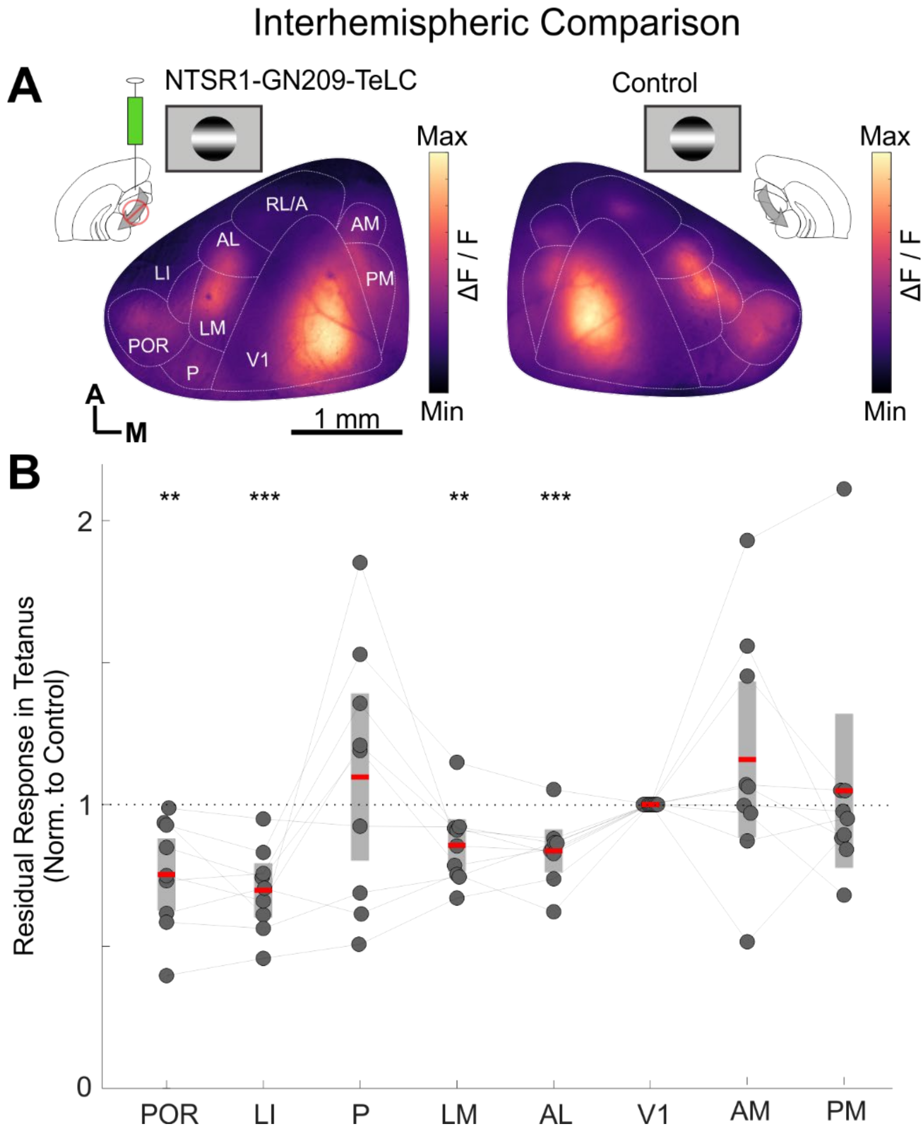

Figure S5. Conditional Expression of TeLC in NTSR1-GN209-Cre mice Reduces Response to Drifting Grating in Lateral Visual Areas, Related to Figure 3.

(**A**) Example ΔF/F across visual areas (5 position, 300 trial average) in TeLC (left) and control (right) hemispheres. Cortical visual areas delineated as shown in Figure S1. Orientation: A: anterior; M: medial.

(**B**) Summary data: Response magnitude for each area in the TeLC-expressing hemisphere relative to the response in the contralateral hemisphere. Within each hemisphere, responses were normalized by the response in their respective V1 (see methods). Gray bar shows 95% confidence interval above and below mean (red line). Gray lines between dots link areas from the same animal. P values are calculated with a t-test (POR: Mean reduction = 24.66%; p = 0.0016; LI: Mean reduction = 30.24%; p = $1.263*10^{-5}$; LM: Mean reduction = 14.38%; p = 0.0075; AL: Mean reduction = 16.30%; p = $6.161*10^{-4}$; N = 9 mice).

1

Figure S6

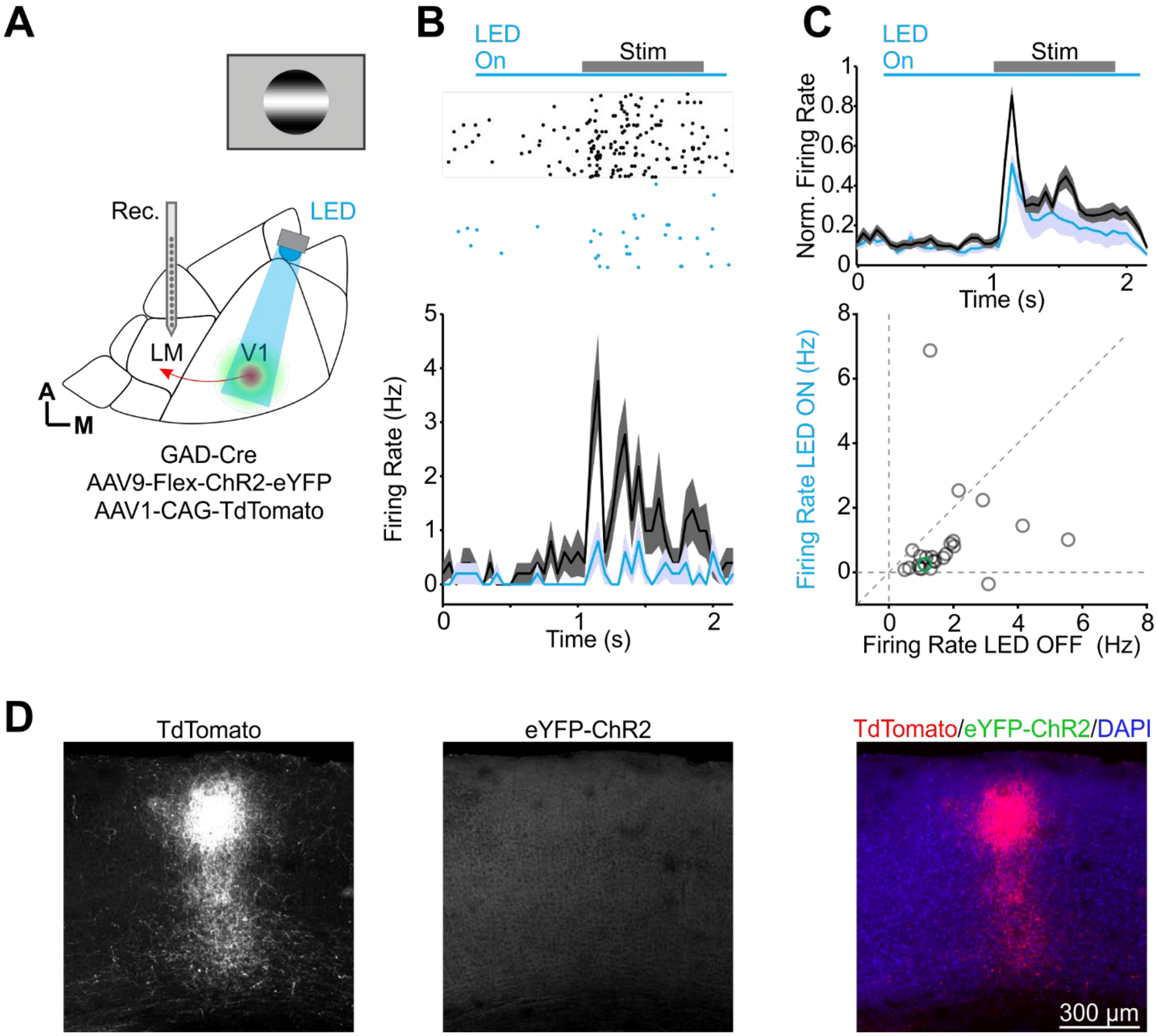
A
Rec.
LED
LM
V1
A
M
GAD-Cre
AAV9-Flex-ChR2-eYFP
AAV1-CAG-TdTomato
B
LED
On
Stim
Firing Rate (Hz)
Time (s)
C
LED
On
Stim
Norm. Firing Rate
Time (s)
Firing Rate LED ON (Hz)
Firing Rate LED OFF (Hz)
D
TdTomato
eYFP-ChR2
TdTomato/eYFP-ChR2/DAPI
300 μm

Figure S6. **Silencing V1 Reduces but Does not Abolish Visual Responses in Lateral Visual Area, Related to Figure 3.**

(**A**) Left: Schematic of experimental configuration: The anterograde red tracer tdTomato injected in V1 enables the identification of the retinotopically matched area in LM (see methods). V1 activity is silenced by optogenetically activating eYFP-tagged ChR2-expressing GABAergic interneurons with an LED coupled to a fiber optic positioned over the tdTomato injection site. As drifting gratings are presented, electrophysiological recordings with a multichannel linear probe are performed in the visuotopically matched location in area LM. This ensures the silencing of the region in V1 that projects to the recorded region in LM. Orientation: A: anterior; M: medial.

(**B**): Raster plot (top) and peristimulus time histogram (PSTH; bottom) of a neuron recorded in LM of an awake head-fixed mouse, in response to drifting gratings (diameter: 20 degrees) under control conditions (black) and during V1 optogenetic silencing (blue). Blue horizontal bar: period of V1 silencing. Gray horizontal bar: period of stimulus presentation. This unit exemplifies a particularly strong suppression of visual responses upon V1 silencing.

(**C**) Top: Summary PSTH for 24 visually responsive regular spiking neurons recorded in LM (N = 4 mice). Blue horizontal bar: period of V1 silencing. Gray horizontal bar: period of stimulus presentation. Bottom: Scatter plot of the visual response of 24 neurons in control conditions and after V1 silencing (Average firing rates, mean ± SEM: LED OFF: 1.76 ± 0.24 Hz; LED ON: 0.87 ± 0.29 Hz; $P<10^{-3}$, n = 24 RS units, N = 4 mice, Wilcoxon signed-rank test). The green data point is the unit in (B).

(**D**) Left: Coronal section (-3.5 mm posterior from bregma) through LM showing tdTomato expressing axons originating from V1. Middle, the absence of ChR2-eYFP fluorescence signal in LM. Right, the merged signals including DAPI staining.

Figure S7

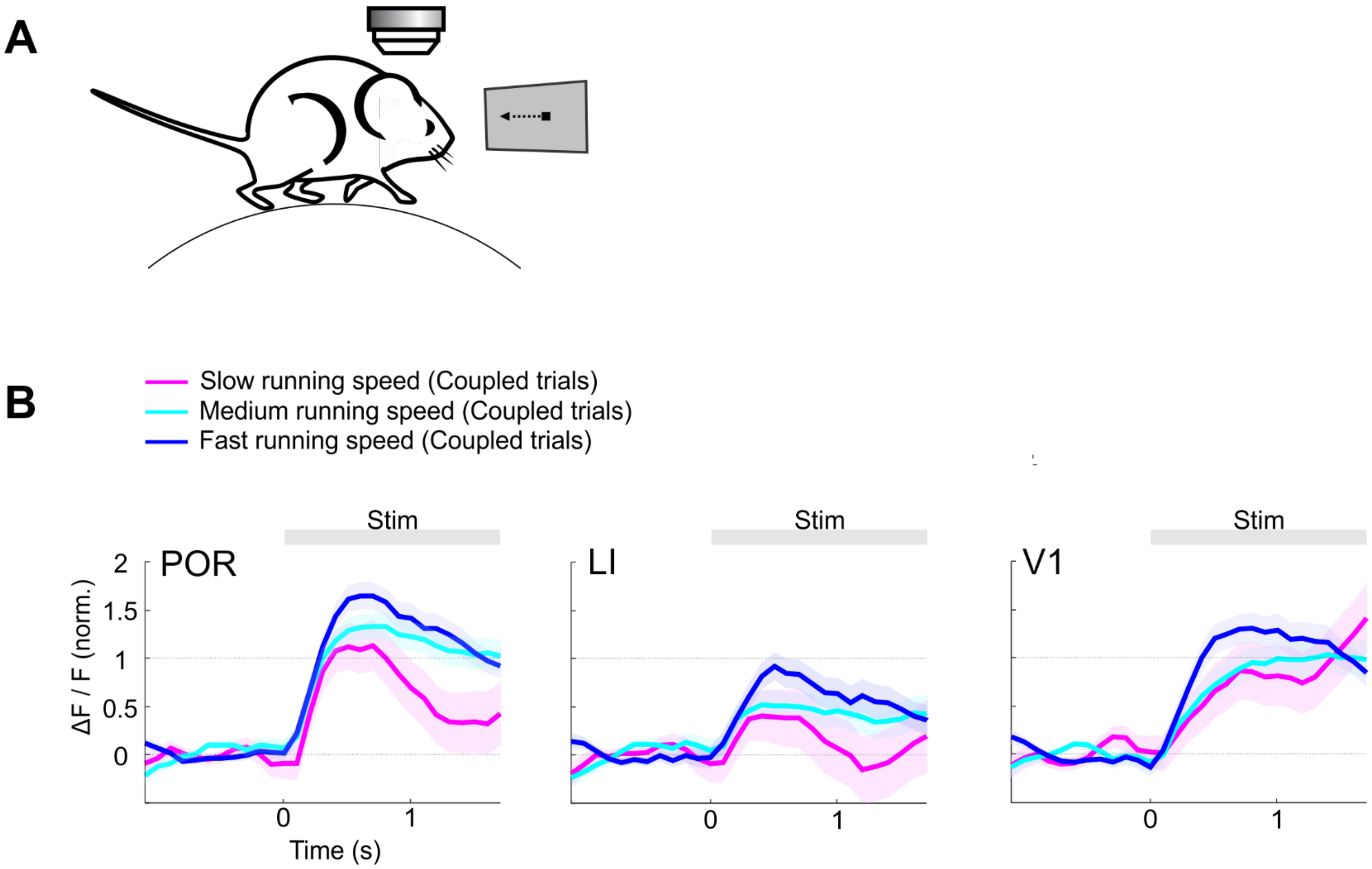

Figure S7. Responses Increase with Running Speed Across Visual Areas, Related to Figure 5.
(**A**) Schematic of experimental configuration for widefield calcium imaging of visual cortex in a mouse running on a treadmill. The mouse is presented with a dot which progresses along a naso-temporal trajectory at a speed commensurate to the running speed of the mouse (coupled).
(**B**) Evoked responses sorted by running speed in three example areas in the coupled condition averaged over all mice (N = 4 mice). Shaded area is SEM. Note that responses increase with running speed across all areas (Pink: Slow running speeds; Green: Medium running speeds; Blue: Fast running speeds).

Figure S8

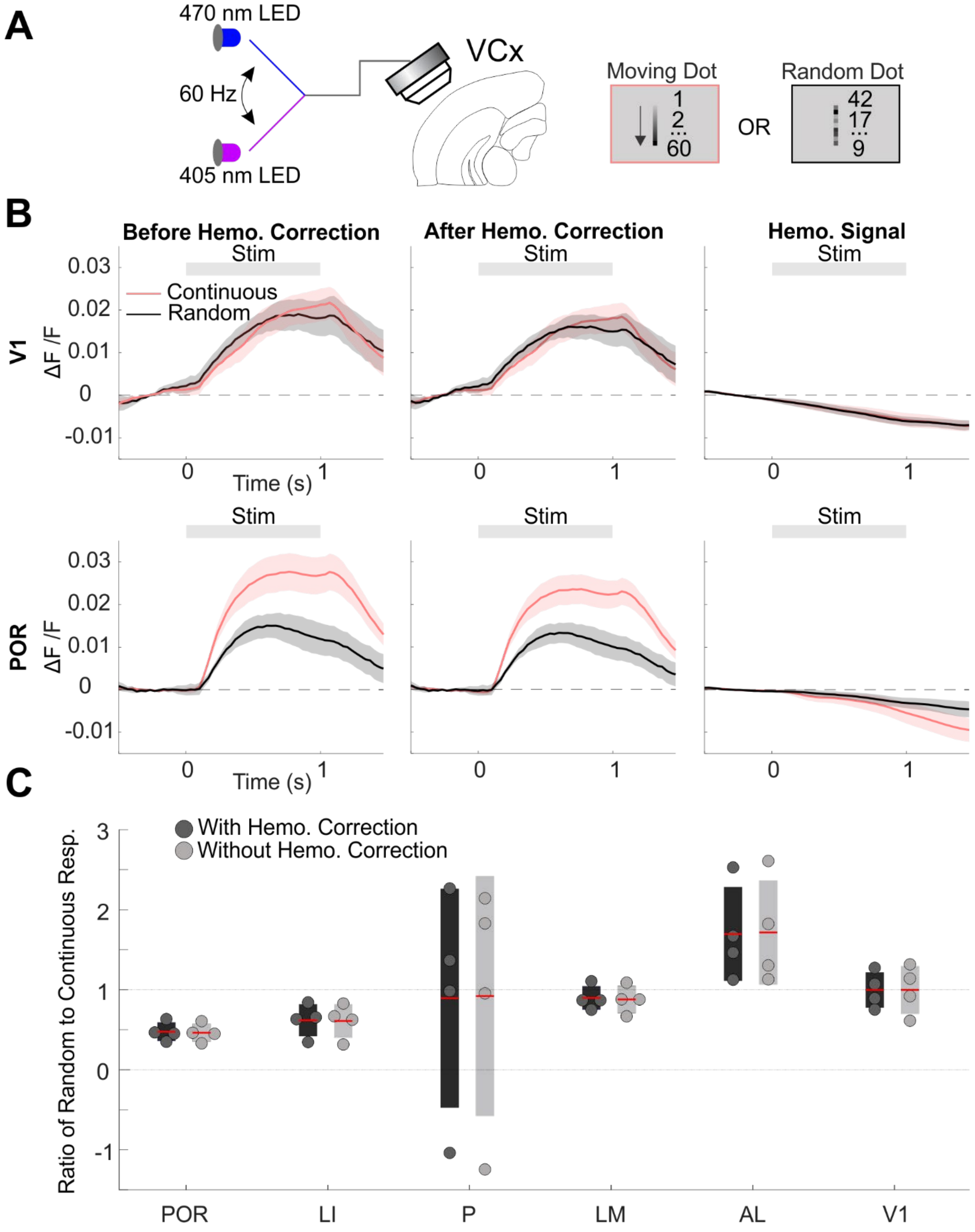
A
470 nm LED
60 Hz
405 nm LED
VCx
Moving Dot
1
2
60
OR
Random Dot
42
17
9
B
Before Hemo. Correction
After Hemo. Correction
Hemo. Signal
Stim
Continuous
Random
V1
POR
ΔF /F
0.03
0.02
0.01
0
-0.01
Time (s)
C
With Hemo. Correction
Without Hemo. Correction
Ratio of Random to Continuous Resp.
3
2
1
0
-1
POR
LI
P
LM
AL
V1

Figure S8. Little Impact of Hemodynamic Correction on Responses to Visual Stimuli Averaged over Large Numbers of Trials, Related to Figure 5.

(**A**) Left: Schematic of setup for hemodynamic correction. Frames illuminated at the approximate isosbestic point for GCaMP6s (405 nm) are interleaved with frames illuminated using blue light (see methods) during widefield calcium imaging of mouse visual cortex. Right: The mouse is presented with a moving dot (2 degrees dark square moving along a 30 degrees vertical trajectory in one second) or with a random dot (same frames used for moving dot but with randomized sequence).

(**B**) Responses of V1 (top) and POR (bottom) to continuous (red) or random (black) dots before (left) and after (middle) hemodynamic correction (average over N = 4 mice; 240 trials each). Raw hemodynamic response is shown on the right. POR but not V1 prefers continuous to random dots, consistent with previous observations (Beltramo et al. 2019). Note similar response magnitude, dynamics and preferences with or without hemodynamic correction.

(**C**) Response to random dot normalized to response to continuous dot for each mouse and area, before (gray) and after (black) hemodynamic correction. Bars show 95% confidence interval above and below mean (red line).
